# Effects of Object Height and AOI Margins on Gaze Measures in a Tabletop Task

**DOI:** 10.3390/jemr19050114

**Published:** 2026-10-05

**Authors:** Fumika Suzuki, Mika Isobe, Kazaru Yaegashi, Yuji Mizoue, Mizuki Nakajima, Yusuke Nagamori

**Affiliations:** 1 Tokyo City University, 3-3-1 Ushikubo-Nishi, Tsuzuki-ku, Yokohama 224-8551, Japanymizoue@tcu.ac.jp (Y.M.); 2 Ritsumeikan University, 56-1 Toji-in Kitamachi, Kita-ku, Kyoto 603-8577, Japan; isobe-m@fc.ritsumei.ac.jp (M.I.); yaegashi@ba.ritsumei.ac.jp (K.Y.); 3 Shibaura Institute of Technology, 3-7-5 Toyosu, Koto-ku, Tokyo 135-8548, Japan; mizuki@shibaura-it.ac.jp

**Keywords:** areas of interest, object height, AOI margin, gaze-to-object mapping, marker-based surface mapping, mobile eye tracking, tabletop tasks, stimulus layout

## Abstract

AOI margin and object height affect gaze assignment; this methodological case study separates them. In a block-imitation task by ten participants, we compared, on the same 4798 fixations, the printed working area (object and support), that area plus a 19 mm margin (separately measured gaze error), and that region raised to object height; the last two differ only in height. Height alone raised the copy area’s dwell share in all ten participants (median +2.4 percentage points), as geometry dictates. For the model’s area, whose region starts on the turntable and can lose gaze, the median was +0.4 points (−5.7 to +32.0), with five rising and five falling. In this layout, the margin moved medians more (copy +7.1, unassigned −14.5). Raised, model and right-tray regions overlapped at every viewpoint; widening them to 40 mm added a median of 0.81 points of assigned time but raised the share qualifying for multiple regions from 0.0% to 13.1%. Two raters judged 200 fixations: the raised region matched their labels more often but also assigned an area where they saw only mat; more assignment is not evidence of accuracy. Size and height are separable choices to report per participant, and their weight depends on layout, tracker and margin.

## 1. Introduction

Eye tracking is often used at a table for tasks in which a person looks at physical objects, picks them up and assembles them. To make such recordings analysable, gaze is mapped onto a plane defined by fiducial markers—a tabletop or a screen [1,2,3,4]—and areas of interest are then drawn on that plane [1,3].

The question asked here comes out of a study of design as it is actually carried out. Nagamori and colleagues had people design with real materials on a table and measured prefrontal haemodynamics during the activity with a wearable functional near-infrared spectroscopy (fNIRS) system, reporting that the material context moderated the association between visualisation and the haemodynamic response [5]. Earlier work in the same line measured frontal haemodynamics during a block-built design task with fNIRS [6], which is where the block task used here comes from. Knowing what a participant was looking at is useful for relating such a physiological change to the visual work context in which it was recorded: checking the model, searching a tray of parts, or assembling. Whether a haemodynamic change differs with the object being looked at is a question that motivates recording gaze alongside it, which is not a result established here. Adding an eye tracker to a tabletop setting of this kind is what produced the problem reported below. This paper does not analyse eye tracking together with fNIRS; it takes up the methodological question that has to be settled before such an analysis can be attempted—how gaze directed at objects standing on a table is to be assigned to areas of interest drawn on the table’s plane.

A homography maps one plane to another [7], so the pipeline reports the point at which the gaze’s ray crosses the marker plane. For a point at height h above the plane, seen from a viewpoint at height C_z and horizontal distance D, that crossing lies further from the viewer than the object stands, by D·h/(C_z − h). An area of interest drawn on an object’s footprint must therefore fail to catch part of the gaze directed at the object, and must do so in one direction. This follows from the projection itself and is not an empirical claim. Whether a particular fixation was in fact given the wrong label is a different, empirical question: the displacement says where gaze directed at the object lands on the plane, not where the participant was looking, and it is settled only by a record of the gaze target that the plane mapping does not provide. What is reported below is therefore of two kinds, and we keep them apart: how far the numbers move when the region is changed, which the recordings establish, and how often the label is right, which they bound only through the scene-video check of Section 3.2. How much the displacement changes the quantities a tabletop study reports has not been established, and neither has how much of that change is due to height rather than to the region simply being larger.

Three bodies of work bear on the question, and the present comparison sits between them. First, for flat stimuli, the consequences of the area-of-interest rule are established: Orquin and colleagues re-analysed four published studies to show which conclusions change with its size [8], and Hessels and colleagues swept size and shape and reported that whether the difference between two regions within a group reached significance changed with the production method, and whether the difference between two groups reached significance changed with the region’s size [9]; how areas of interest are defined sits among the threats to validity catalogued for eye-movement research more generally [10]. The current synthesis recommends testing this for one’s own data and using a margin at least as large as the measurement inaccuracy, and it illustrates the problem directly: Figure 9A–C of [11] shows a marker-covered table on which two people built a LEGO model, and notes that a gaze point lying on a hand in the overhead view appears to lie underneath the hand in the scene camera “because the gaze points are projected onto the plane of the table” [11]. The displacement is therefore recognised. Second, the accuracy of the tracker and of the mapping has been measured, but as a whole and against targets on a plane: published evaluations of the tracker used here, two of them independent of the manufacturer, report inaccuracies of 1.4° to 2.4° [12,13,14], with one of them having the same cloud surface mapping [12]; a standard procedure exists for measuring the figure per participant [15], and how it is to be determined and reported is the subject of standing guidance [16]. Where gaze has been checked against an independently measured target in three dimensions, it was by integrating the tracker with motion capture [17,18], which bypasses the marker plane rather than testing it. Third, work that takes real objects seriously builds alternatives to the plane: geometric proxies with ray-casting [19], photogrammetric models [20], segmented object masks [21], and motion capture synchronised with the eye tracker to generate gaze vectors in three dimensions [18]. None of these reports what the planar rule costs; Jensen and colleagues place plane- and 3D-mapped gaze side by side and state that the two “cannot be compared directly” [22]. The closest case to ours varies the size of the analysis window with the distance to the object and compares the result with a fixed size, but on scanpath image similarity rather than on dwell or assignment [23]. Two further distinctions run through this literature and through the present paper. A study can show that a rule assigns more gaze, or that it assigns gaze correctly, and the two are not the same: Wang and colleagues vary the assignment criterion on real wearable recordings and raise the share of interpretable fixations for one of two objects from 23.8% to 78.3% when the criterion is widened from a region hit to the parafoveal field of vision, while cautioning that processable data is “not equal to accurate” [24], whereas the simulation of Vehlen and colleagues, in which the target is known, shows a wider region admitting gaze that belongs to a neighbouring feature [25]. And where the check is a human reading of the scene video, what it measures is the reproducibility of that reading between raters [26,27,28,29], not the correctness of the gaze estimate. How far apart the resulting measures lie, when a flat and a height-aware definition are given the same margin on the same fixations, has not, to our knowledge, been reported.

We therefore ask a narrow question, on a tabletop task and with no additional hardware: on the same fixations, how far apart are the dwell shares, the unassigned share and the transition rates produced by a flat area of interest and by one that carries the object’s height, when both are given the same margin?

## 2. Methods

### 2.1. Task, Objects and What the Participant Did

**Figure 1:**
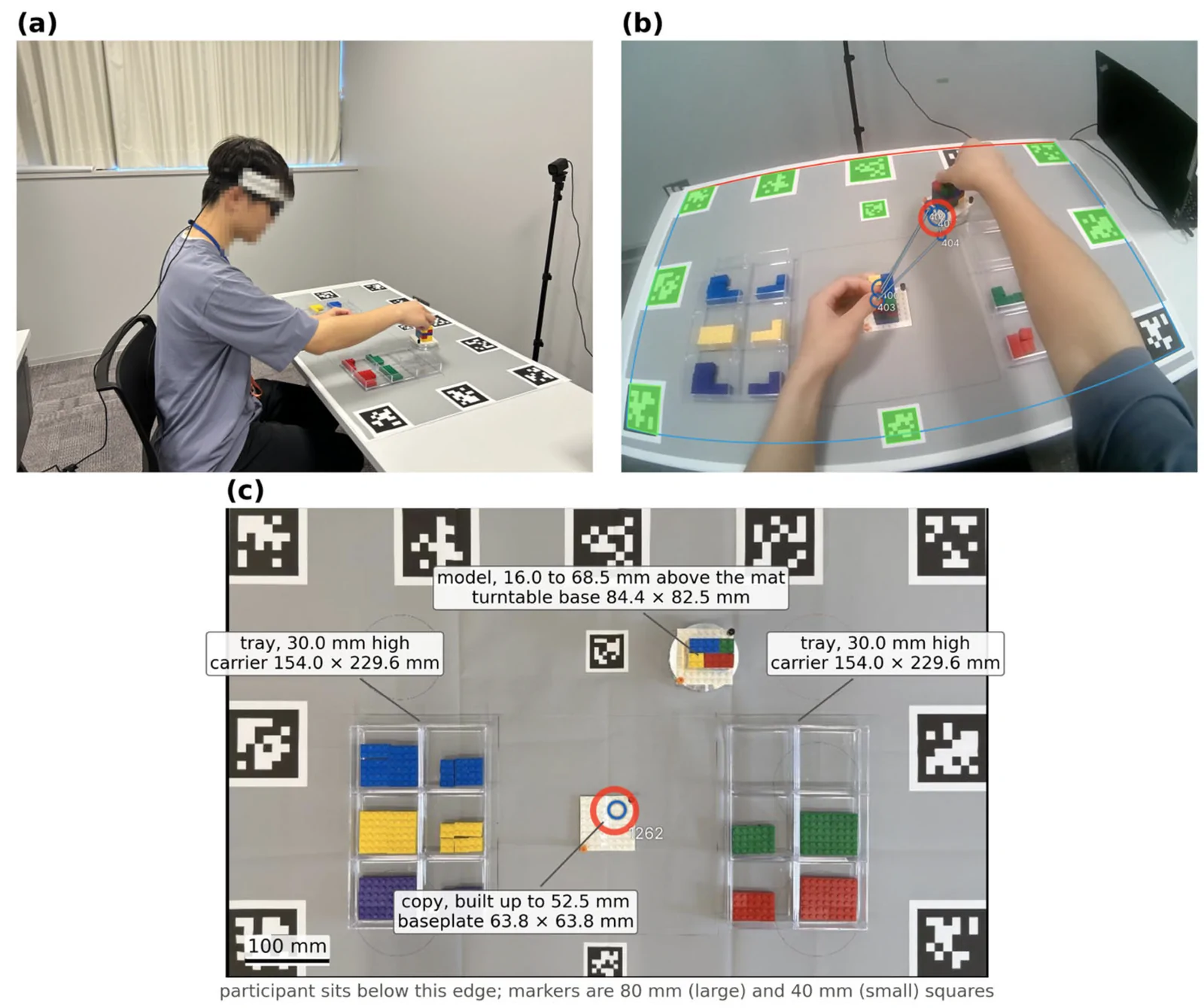
(**a**) A participant at the mat, seated, wearing the eye tracker and the forehead probe of the wearable fNIRS device used in the parent study; their face is obscured. The objects stand on the table in front of the participant, and the viewing angle to their upper surfaces is oblique. (**b**) A scene-camera frame from an imitation block with the enrichment’s overlay: the decoded markers, the outline of the localized surface, and the gaze estimates with their fixation numbers; only the participant’s hands and forearms are visible. (**c**) The enrichment’s reference image of the mat: the model on its turntable (upper centre), the baseplate on which the copy is built (centre), and the two trays of bricks (left and right), with the printed size of each carrier and the object’s height written beside it (the values of Table 1) and an approximate 100 mm scale bar derived from the 80 mm markers; the participant sits beyond the lower edge, and the large and small markers are 80 mm and 40 mm squares. The circle labelled 1262 in (**c**) is a fixation marker drawn by the enrichment’s overlay when the image was exported and is not part of the layout.

Eleven students were recorded in a seated session on a printed mat carrying eleven AprilTag markers [30] of the tag36h11 family around the working regions (nine with an 80 mm black square and two with a 40 mm one; Figure 1c). One is not analysed: for that recording, the Marker Mapper enrichment ended with an internal server error in Pupil Cloud and returned no output, so it carries no surface coordinates at all. The cause of the error could not be determined, so we cannot rule out that something in the recording contributed to it; the exclusion follows from the missing output, not from a judgement of recording quality or of the participant’s behaviour. No criterion of recording quality was applied before the sample was fixed, and no fixation was removed for quality beyond the requirement of Section 2.3 that it lie on the localized surface. Ten participants remain, aged 18 to 24 (median 22), all right-handed, all students; the sample is small and homogeneous, and the study is a methodological case study on it, not an estimate of a population. They were recruited at Tokyo City University and at Ritsumeikan University and had no part in the research; none of them were compensated. The eye tracker’s frame accepts corrective lenses: one participant chose the lens that suited them from the manufacturer’s lens set at the start of the session and wore it throughout, and the other nine wore the clear lenses the frame carries by default. Each of them performed one session of four blocks in a fixed order: two of a block-imitation task, a physical version of the block-copying paradigm [31], followed by two of a free-design task. The order was fixed because the free-design task is the one that the parent study is about [5], and the imitation task was placed before it as the standardised task of the session; the two imitation blocks were therefore the first and second blocks every participant performed, at the start of the session rather than after it. Two limits follow. The order was the same for every participant, so an order effect—how the same choices of region would behave for an imitation task performed after another task, or later in a session—was not tested. And a comparison of the two imitation blocks (Section 3.1; Supplementary Material S1–S10) can show whether the numbers changed between the first and the second block, not why: practice, fatigue and the counterbalanced layer order of the model are confounded in it. Only the twenty imitation blocks are analysed here; the free-design blocks enter no Figure or Table below, except that the poses recovered during them contribute to each participant’s single viewpoint (Section 2.2). Sequential object interaction was studied with eye tracking before, in a fixed-sequence bimanual cup-stacking task at a table [32] and, with the hand tracked alongside the eye, in standardised object-transfer tasks [33].

The model is a solid structure made of twenty plastic bricks (ten 2 × 4 and ten 2 × 2, in five colours used in equal numbers), stacked five layers high on a footprint of 6 × 4 studs. It stands on a small manual turntable at the far side of the mat and cannot be lifted: to see a face turned away from them, the participant pushes the edge of its plate and turns it. The bricks to build with lie in two transparent trays, one to the left and one to the right, with each colour and shape in its own compartment, in a layout that was the same for every participant. The participant takes bricks by hand and assembles a copy on a baseplate directly in front of them (Figure 1c). At the start of each block, they were asked to build so that it would be the same as the model; the working rules had been given in a practice trial beforehand: fix the bricks to the baseplate, build upwards from the bottom, turn the model rather than lift it, and put accuracy before speed. No time limit was set and no clock was shown, and a block ended when the participant said they had finished. Each participant built two models that differed only in the order of their layers, one per block, with the order counterbalanced, with five participants each way. The twenty blocks analysed here ran for a median of 130 s (86–274 s).

Gaze therefore moves between an object the participant only looks at, objects they reach into, and an object that grows under their hands; these are the model, resource and workspace regions of the copying paradigm, here as real objects with height. The four objects differ in height and in distance from the participant, so a single layout supports the comparison made below.

Each object stands on a carrier whose position is printed on the mat, so the horizontal extent of every region is known from the print file and is held fixed throughout. Heights were measured for the turntable, the model, and the trays (Table 1). The turntable base is 16.0 mm, and the model on it is 52.5 mm, so the model occupies 16.0 to 68.5 mm above the mat; the trays are 30.0 mm. The copy is a different case. Its height changes as it is built and we did not measure it; the participant reproduces the model, so we applied the finished model’s measured height to the copy throughout each block, taking it to occupy 0 to 52.5 mm above the mat. That is a proxy for a changing quantity, and it errs in one direction: early in a block the copy is lower than the proxy, so the raised region then extends further from the participant than the object does and can claim gaze mapped beyond the copy, towards the model, that the object itself would not have caught; late in the block the proxy is close to the true height. How much this matters is bounded in Section 4.2 from Supplementary Material S3b, which repeats the main analysis with the proxy set to 24 and 96 mm; a height that followed the build brick by brick would have to be coded from the video and was not attempted. S3a varies the same assumption on the per-frame variant. Table 1 also gives the displacement D·h/(C_z − h) that follows for each object from these heights and the viewpoints recovered below. The regions and the viewpoints are expressed in the same coordinate frame, and each participant contributes one value, computed from that participant’s single viewpoint; the Table reports the median over the ten participants and the range. Because the viewpoint is one per participant rather than one per task, the model’s figures use the same viewpoint as the rest.

### 2.2. The Regions Compared

**Figure 2:**
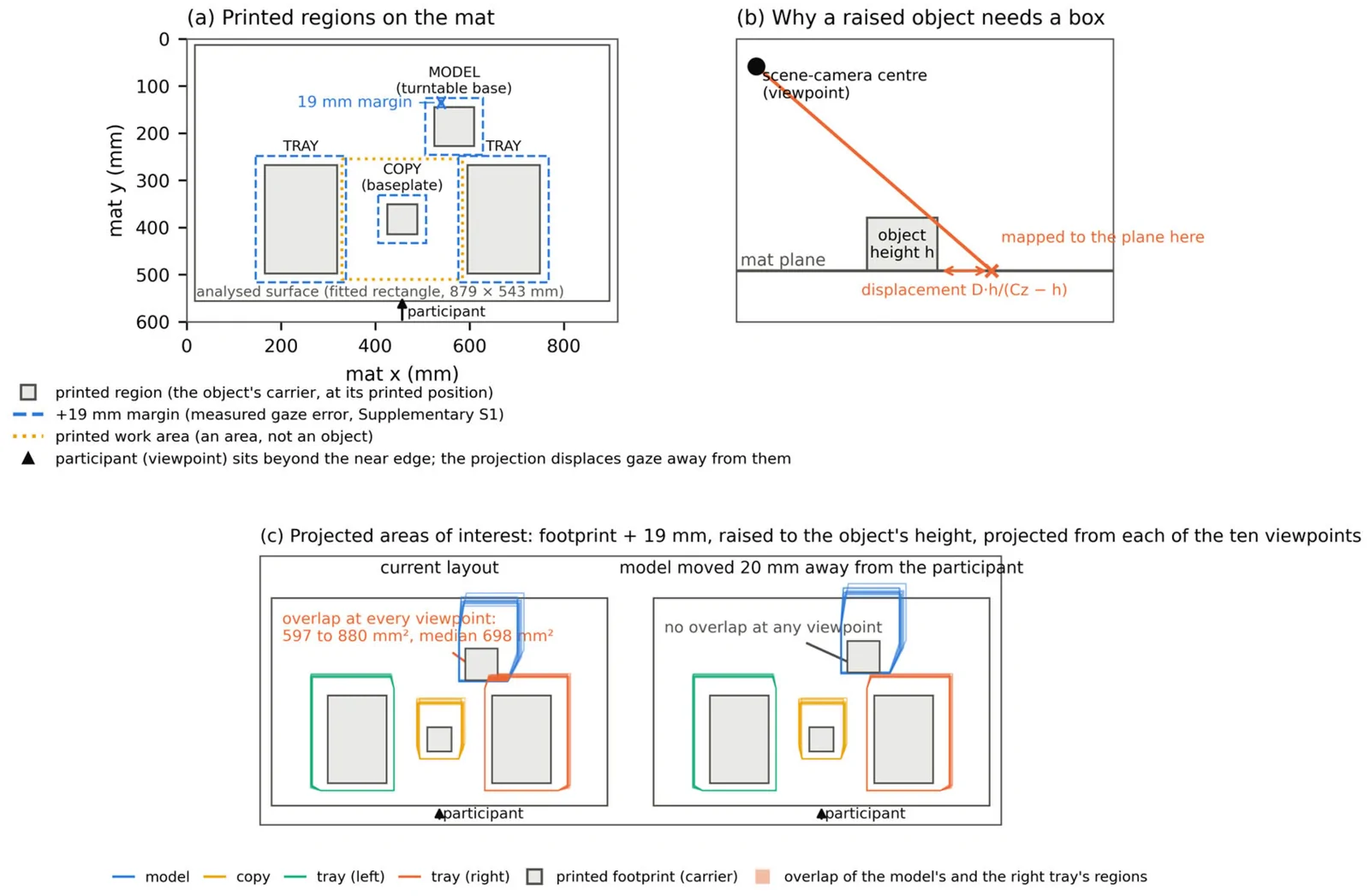
(**a**) The printed regions on the analysed surface, the 19 mm margin, and the printed work area; the arrow marks the edge beyond which the participant sits, so the projection displaces gaze upwards in this view, away from them. (**b**) Why a raised object needs a region with height: the line of sight to the top of an object of height h crosses the mat plane beyond the object’s footprint, by D·h/(C_z − h). (**c**) The same regions—each printed footprint (grey) enlarged by the 19 mm margin, raised to the object’s height and carried to the mat plane by that projection—have one outline per participant viewpoint, for the imitation task. Left: the layout used here; the model’s and the right tray’s regions overlap at every viewpoint (filled; 597 to 880 mm^2^ across the ten viewpoints, median 698 mm^2^, Section 3.3). Right: the same layout with the model moved 20 mm away from the participant, the smallest move on the 5 mm grid that leaves no pair overlapping at any viewpoint at the 19 mm margin. Computed from geometry alone; no fixation enters this panel.

Three regions are compared. All three use the same printed extents and the same fixations, so only one factor changes at a time (Figure 2).

- Flat. The fixation is assigned to an object’s area if its mapped position lies inside that object’s printed region.
- Flat + 19 mm. The same, with every region enlarged by a margin on all sides.
- Raised + 19 mm. The same enlarged region, but the fixation is assigned only if the segment from the viewpoint to the mapped position passes through the region raised to the object’s height.

Each region is a working area rather than an object: its printed extent is that of the carrier the object stands on—the turntable for the model, the baseplate for the copy, the tray itself—so a fixation assigned to a region may have been on the object, on its carrier, or on the mat near them (Section 3.2). Below, where a share or an assignment is reported, the model, the copy, and the trays name these areas, not gaze verified on the objects themselves (Section 4.2).

**Figure 3:**
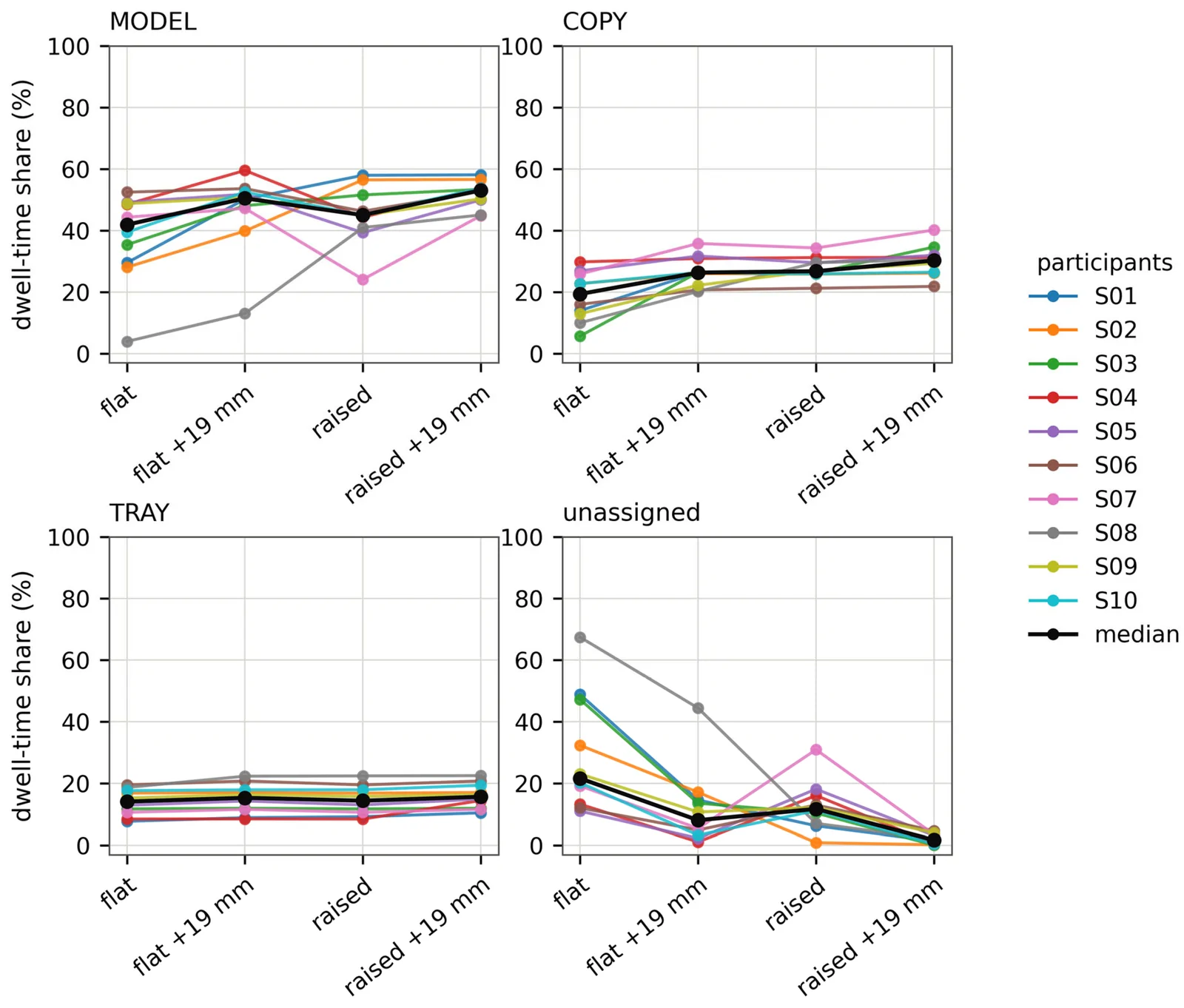
Dwell-time share under each region, one line per participant (*n* = 10); each participant keeps the same colour and label (S01–S10, as in Supplementary Material S9) in every panel, and the black line is the median of the ten participant-level shares at each region. “Flat” is the printed region on the plane, and “raised” is the same region carrying the object’s height. MODEL, COPY, and TRAY are the working areas of Section 2.2, with each object having a carrier it stands on, and “unassigned” is the share assigned to no area.

The fourth region, raised with no margin, is the printed extent raised to the object’s height. It is not part of the comparison—neither contrast of interest uses it—but it is shown in Figure 3 so that the two factors can be read in either order: flat to flat + 19 mm isolates the margin at zero height, and raised to raised + 19 mm isolates the same margin once the regions carry height. Where a fixation qualifies for more than one object, it is assigned to the box the segment enters first. The height contribution reported below therefore depends on two things at once: on the geometry of each object’s box relative to its printed region, which decides what the raised region can gain and lose, and on how that tie-breaking rule resolves the overlaps between raised regions that this particular layout produces; Section 3.1 separates the two, and neither is a property of height in general.

The comparison that isolates height is therefore raised + 19 mm against flat + 19 mm, in which the regions have the same footprint and the same margin and differ only in whether they carry the object’s height. The comparison of flat + 19 mm against flat isolates the margin. We report both, and we do not describe a difference in which both changed as an effect of height.

The printed positions used throughout are the nominal coordinates of the mat’s design file, not positions measured after printing; the enrichment recovers the surface from the markers as printed, so a scale error in printing would enter both the regions and the mapped gaze in the same way. The margin is 19 mm because that is the median gaze error measured for this setup in a separate session, worn by one of the authors rather than by a participant (19.0 mm over the eleven targets and 1.79° at the distances of that session, with a median precision of 5.6 mm or 0.47°; 22.0 mm over the nine evaluation targets alone; Supplementary Material S1), following the recommendation that a margin be at least the measurement inaccuracy [11]; regarding how such a figure is to be determined and reported, see [16]. A per-participant figure obtained with a standard procedure such as glassesValidator [15] would be preferable to a single session, and we did not collect one. Nothing below makes 19 mm an optimal or a generally applicable value: the margin a study needs depends on its participants’ own gaze accuracy, on the viewing distance and angle, and on the tracker and its configuration, and 19 mm is simply the value this setup measured once. Published evaluations of the same tracker report a mean inaccuracy of 1.45° with the same Pupil Cloud surface mapping at a viewing distance of 60 cm, with the reference tracker’s infrared source occluded [12], 1.41° with the head supported and a one-point calibration at 65 cm [13], and an effective 2.1° to 2.4° without a user-specific calibration during everyday activities [14]; at the line-of-sight distances of Table 1, those figures span 11 mm (1.41° to the copy) to 25 mm (2.4° to the model), so the 19.0 mm used here sits inside the range they imply. The single margin is not equally tight on every object: 1.79°, the angular equivalent of the 19.0 mm at the distances of the measuring session, corresponds to 18.6 mm at the model’s line-of-sight distance but 14.3 mm at the copy’s, so a uniform 19 mm is slightly generous for the copy and about right for the model. The 19.0 mm is a distance on the mat, which is viewed obliquely, so its angular equivalent is smaller than a conversion at normal incidence would give. That figure rests on one measurement session, so we repeated the whole comparison with the margin varied from 14 to 24 mm in 1 mm steps; what that sweep gives is reported in Section 3.1, and in full in Supplementary Material S1b. Two quantities also change with the margin and are reported in Section 3.3: how much the projected regions overlap, and how far an object has to move to clear that overlap. The layout also sets a ceiling on the margin: the model’s and the right tray’s printed regions are 40.3 mm apart, so above about 20 mm, the flat regions themselves begin to overlap and the flat rule starts resolving ties.

Because the viewpoint is fixed within a participant, the raised region can also be written as a region on the mat plane: the central projection of the box from the viewpoint onto the plane, which for a box is the convex hull of the images of its eight corners (Figure 2c). We use this planar form only to consider layout, and with the same convention as the assignment rule: enlarge the footprint by the margin, then raise it, then project. Projecting first and enlarging in the plane afterwards is a different rule and is not used here. On all 4798 analysed fixations, the planar form agrees with the segment-and-box test used for the assignment, for every object and every fixation, with no exception. Which object a fixation is finally given is a separate matter: where two projected regions overlap, the rule assigns the box that the segment enters first, so an overlap is an ambiguity in the layout rather than in the rule.

To ask whether a layout could be changed to remove such an overlap, we translated either of the overlapping objects on a 5 mm grid over ±80 mm in x and y, subject to keeping every object on the mat and the printed footprints not overlapping physically, and it was required that no pair of projected regions intersect at any of the ten viewpoints. This is geometry only: the viewpoints are held at the ones recovered from the recordings, and no gaze is re-applied to a changed layout.

The viewpoint is the scene camera’s optical centre, recovered from the four exported surface corners by perspective-n-point using the camera parameters exported with each recording. One viewpoint per participant is used: the median of that participant’s accepted poses over the whole recording, including the free-design blocks. A per-frame alternative, the pose recovered for each frame separately, can be compared with it only on the 2498 fixations whose pose was accepted, a subset of the analysed set that is unevenly spread over the participants: it holds 52 to 450 fixations per participant, which is 10% to 98% of each participant’s analysed fixations (S10c). What that comparison gives is reported in Section 3.1 and in full in Supplementary Material S2. We therefore report the fixed viewpoint, noting that the difference was small where the two could be compared, not that the measures are independent of viewpoint over time.

The candidate objects are the model, the copy and the two trays.

### 2.3. Recording, Processing, the Analysed Set and the Measures

Gaze was recorded with a Pupil Labs Neon wearable eye tracker (Pupil Labs GmbH, Berlin, Germany) in the “I can see clearly now” frame, binocular at 200 Hz, with one module for all ten participants; the Companion app was 2.9.47-prod, gaze pipeline 2.9.0, and firmware 29.8. The app’s gaze offset correction was applied only where the gaze marker was visibly displaced at the start of a session, not uniformly to every participant. Which participants received it, and by how much, is recorded in each recording’s metadata (the gaze_offset field of info.json): six of the ten received a correction, of 5.7 to 96.8 px in the scene camera, which is 0.4° to 6.2° at the image centre, and four received none (S9, S10e). The app applies the correction to the gaze estimate as it records, so every analysis below contains it; what it does not provide is the error that remained after correction, which was not measured for any participant. A correction therefore says how far a participant’s gaze was shifted, not how accurate it was afterwards, and its size is not used below as a measure of that participant’s accuracy. Recordings were uploaded to Pupil Cloud and processed with its Marker Mapper enrichment (export version 2) [1], which detects the markers, localizes the mat as a planar surface in each scene-camera frame, and re-expresses gaze in coordinates normalized to that surface. Fixation detection is Pupil Cloud’s, not ours: fixations are those returned by the cloud’s own detector for the Neon, which is designed for head-mounted recording and compensates for head movement, including the vestibulo-ocular reflex, and which operates before the mapping onto the surface rather than on the mapped coordinates [34]; we applied no detection, filtering, or smoothing of our own. The detector has no user-settable parameters, and every recording was uploaded to the same workspace and processed with the same enrichment settings and the same export version; the Companion app, gaze pipeline, and firmware versions given above are those written into every one of the ten recordings’ metadata. Neither the recordings’ metadata nor the enrichment export gives a separate version number for the fixation detector: the versions above are those of the app, the gaze pipeline, and the firmware, not of the detector, which is the one described in the documentation cited [34]. The definition of a fixation is therefore the detector’s; definitions differ across the field, and they differ most where the head is free and the vestibulo-ocular reflex is present, which is the case here [35]. The analysed table was built from two exports. The fixation series, with each fixation’s identifier, start and end timestamps, and duration, comes from the recording’s own fixation export; the Marker Mapper export is joined to it by recording and fixation identifier and adds whether the fixation was detected on the localized surface and its position in surface coordinates. The transition analysis uses adjacency in that original series, so two fixations count as adjacent only if they were adjacent in the recording. Two further exports are used in Section 2.2: the surface corner positions written for each frame, and the scene camera’s intrinsics and distortion coefficients written with each recording.

Task windows were taken from onsets coded from the scene video. Of the 5251 fixations inside the imitation blocks, 4798 (91.4%) fell inside the localized surface and are the set analysed under every region. No region is given a different set of fixations than another. The 453 fixations that did not reach the surface are not spread over the task: 403 of them lie in the last third of a block, and 407 belong to five participants, with 397 of them in the last third of a block, where they make up 17% to 68% of that third’s fixations (S10d); three other participants have theirs mostly in the first third, and the middle thirds of all twenty blocks together hold six. Whether the participant looked away from the mat or the surface was not localized in the frame was not coded, so these fixations enter no region under any definition, and the dwell shares below are shares of the time that did reach the surface. S10f repeats the main contrasts on the first two-thirds of the blocks, where nearly every fixation reached the surface, and with the participants divided by coverage (Section 3.1).

Dwell share is the fixation time assigned to each object’s area as a percentage of the analysed fixation time in that task, that is, of the time that reached the localized surface, with the unassigned share included so the figures sum to 100. Transition rate counts pairs of fixations adjacent in the raw sequence, within the same block, both assigned to working areas, with different labels and a gap of at most 1 s, divided by task time. The 1 s limit is there to keep a pair from spanning a stretch without fixations, such as a blink, a look away from the mat or a loss of tracking; of the 4737 evaluable adjacent pairs, it removes 13 (a limit of 0.5 s would remove 41 and a limit of 2 s would remove 3), and none of the pairs removed at any of these limits is one of the two reported pairs under any of the three regions compared, so the rates are the same with the limit at 0.5 s, at 2 s or removed (S10b). The count is directed: a pair is counted for the ordered pair of labels it carries, so model to copy and copy to model are separate. We report two ordered pairs, model to copy and tray to copy, because the imitation task is expected to produce them in that direction—the first when the model is checked before a brick is placed, and the second when a brick taken from a tray is brought to the copy; they are counts of gaze moving between areas, and whether a brick was in fact placed or picked at a given transition was not coded. The remaining ordered pairs are in the archive.

Each participant contributed two blocks, and the two are pooled within the participant: a dwell share is computed over the fixations of both blocks together, and transitions are counted within a block (a pair never crosses a block boundary) and divided by the summed duration of that participant’s two blocks. Participants, not blocks, are the unit of analysis. Every contrast is computed within participants and reported as the median of those differences with its range and the number of participants on each side of zero. Differences between group medians are not reported as within-participant effects.

**Figure 4:**
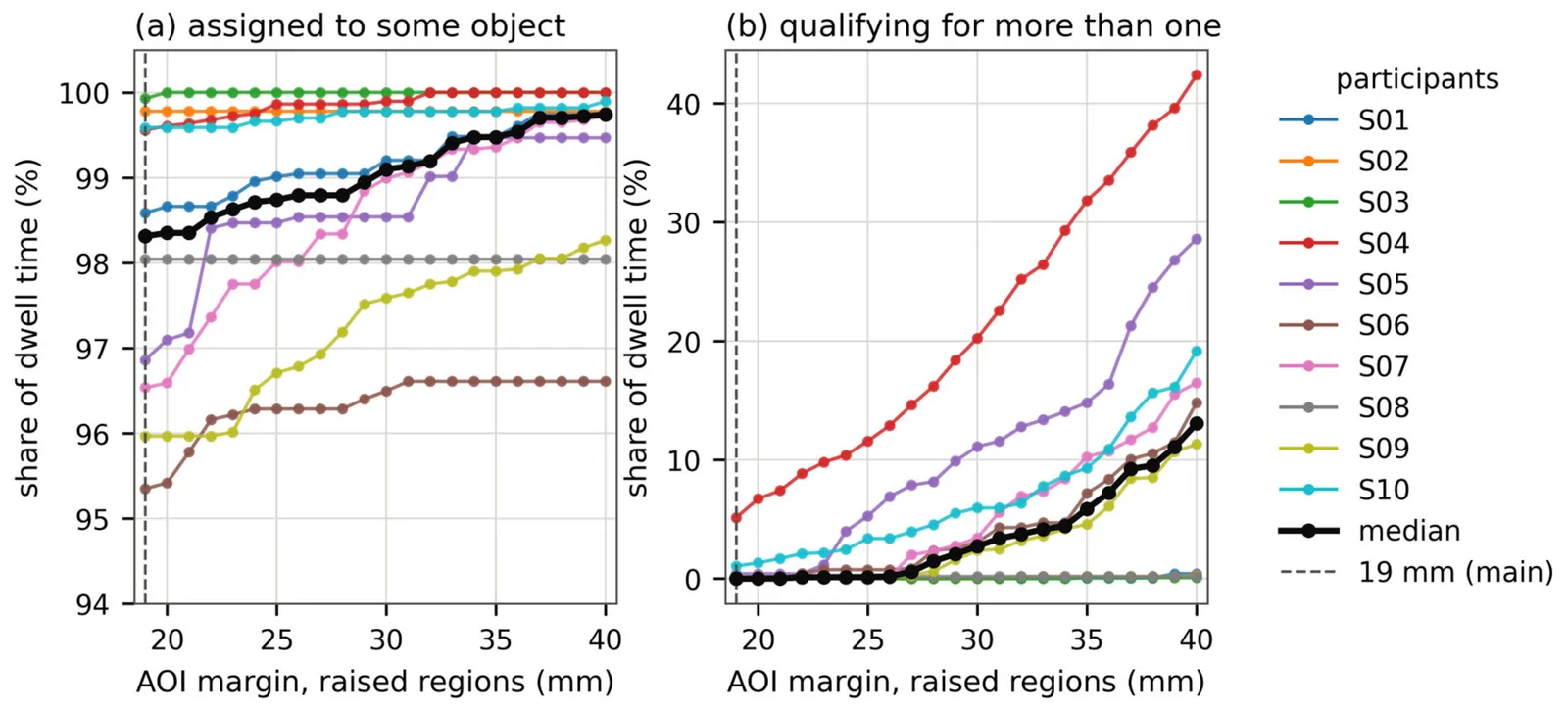
Widening the margin on the raised regions from the 19 mm used in the main analysis to 40 mm, on the same 4798 fixations and the same per-participant viewpoints. (**a**) The share of dwell time assigned to some object’s area. (**b**) The share of dwell time whose fixation qualifies for more than one region, counted before the tie-breaking rule is applied. One line per participant (n = 10), with the same colours as Figure 3; black is the median over participants at each margin; the two panels share the horizontal axis, and the dashed line marks the 19 mm of the main analysis. The heights, the printed footprints, and the tie-breaking rule are those of the main analysis, and only the margin changes. The height contribution over 14 to 24 mm, which this Figure does not address, is in Supplementary Material S1b.

Widening the margin on the raised regions. For Section 3.1 and Figure 4, the margin applied to the raised regions was varied from 19 to 40 mm in 1 mm steps, with the same 4798 fixations, the same per-participant fixed viewpoint, the same object-height assumptions and printed footprints, and the same overlap-resolution rule as the main analysis; only the margin changed. For each participant and each margin, we computed, over that participant’s analysed fixation time, the share falling in at least one region and, with the same denominator, the share entering two or more regions before the overlap-resolution rule is applied. Both are shares of fixation time, not of the number of fixations. Figure 4 plots the individual curves and the median over participants at each margin.

A check against the scene video. From the 4798 analysed fixations we drew 200 for a check against the scene video: twenty per participant, stratified by block and by whether the fixation fell in the first, middle or last third of that block, under a fixed random seed. No region’s labels entered the sampling, so fixations that the rules disagree about are not over-represented; a total of 24 of the 200 (12.0%) are labelled differently by the flat region with a margin and by the raised one. Each item shows the scene frame with the gaze marker, a 520 px crop around the marker at its original scale, and the frames 200 ms before and after, with the 200 items shuffled so that the sheet number does not identify the participant. Two members of the laboratory, neither of whom had seen any assignment under any region or knew which region definition was being evaluated, independently named what the centre of the marker was over, choosing one of seven labels: the model, the copy under construction, either tray, a bare part of the copy’s baseplate, the turntable and the white board beneath the model, the mat surface, or unclear. They separately flagged the items where the marker was covered by a hand, or by a brick the hand was holding. The two judged the same 200 sheets in the same shuffled order, each in a single pass, without conferring and without seeing the other’s responses, and neither took part in writing the assignment code or in the analysis; their responses were exported separately and combined only after both were complete.

Two conventions are needed to compare those labels with the regions, and both are applied throughout Section 3.2. First, the seven labels are folded onto the four areas of interest: a bare part of the baseplate counts as the copy’s area, and the turntable and the board beneath the model count as the model’s area. The regions are drawn on printed footprints, and the printed footprint of the model *is* the turntable, so a fixation on the turntable is inside the model’s area by construction; the fold makes each label a working area rather than an object body, and Section 4.2 returns to what that costs. Second, items are excluded per rater: where a region is compared with one rater’s labels, that rater’s own covered and unclear items are dropped, leaving 171 items for the first rater and 176 for the second. The agreement between the two raters is computed on the 167 items that are usable for both, that is, with the covered items of either rater and the unclear items of either rater removed, and is reported as percentage agreement and as Cohen’s κ [36]. Figures with the covered items kept (196 items between the raters and 198 for each rater against the regions, with the unclear items still removed) and under the other three ways of folding the labels are in S4, and are not the figures reported below.

## 3. Results

### 3.1. At the Group Median, the Margin Contributes Most: The Height Contribution Varies Widely Between Participants

Table 2 gives the three regions side by side, with the two contrasts separated. Figure 3 shows every participant. All Figures below are within-participant differences summarised as the median over the ten participants, with the range in brackets.

Enlarging the flat region by the measured gaze error accounts for most of the change. It raises the copy’s share by +7.1 points [1.1, 20.7] and the model’s by +10.1 points [1.2, 20.7], and lowers the unassigned share by −14.5 points [−34.2, −7.2]; all three are in the same direction in all ten participants.

Giving the same enlarged region the object’s height adds a further amount. At the group median this is about a third of what the margin gave for the copy, but the ranges in brackets matter more than the median: +2.4 points [0.1, 10.3] against the margin’s +7.1, with all ten participants increasing. The unassigned share falls by a further −4.8 points [−42.5, 0.9], with nine participants decreasing and one increasing. The trays, which are low and wide, move by a median of 0.1 points once the margin is in place, with six of ten on the positive side.

On the model, the group median is near zero, but the participants are split. The median contribution is +0.4 points [−5.7, 32.0], with five participants increasing and five decreasing, even though the model is the tallest object and has the largest displacement (Table 1). The small median does not mean that height made no difference to these recordings: one participant’s model share moved by 32.0 points on height alone, while another’s moved by −5.7. The group median therefore did not capture the opposing changes across participants.

Decomposing the change makes the split interpretable. In the present layout at the 19 mm margin, the copy’s raised region contains its flat one, because its box stands on the mat, and its projected region overlaps no other object’s (Section 3.3). Its assignments can therefore increase but cannot be lost to another object’s area under the tie-breaking rule: all ten participants gain between +0.1 and +10.3 points, and none loses anything (Supplementary Material S7). The model’s region begins at the top of the turntable, 16.0 mm above the mat, so that a strip of the flat region nearest the participant falls outside the raised box: the line of sight reaches the mat before it can cross the box’s near face. That strip runs from the near edge of the enlarged flat region—the printed edge plus the 19 mm margin, at y = 233.6 mm in surface coordinates—back to between y = 214.0 and 219.2 mm depending on the viewpoint, a width of 14 to 20 mm. It is a thin band at the near edge of a region that holds 1707 of the flat rule’s assignments, and only 76 fixations were mapped into it at all. Because the band’s far boundary moves with the viewpoint, 18 of those 76 fall inside the widest band but still cross their own participant’s box, so 58 are lost; that is also why the losses span a narrower range of y than the band drawn over all ten viewpoints. The model’s 95 lost fixations, counted over all ten participants, divide accordingly: 58 lie in the strip, of which 47 enter no box at all and become unassigned and 11 enter only the right tray’s box, while the remaining 37 enter both boxes and go to the tray under the tie-breaking rule. Those 37 are the fixations that fall in the projected overlap reported in Section 3.3, so the two counts describe the same geometry from either side. The 241 fixations that the model gains, again summed over the participants, lie further away, between y = 5.0 and 197.2 mm, and every one of them was unassigned under the flat rule.

The split across participants follows from whichever of the two dominates. The five whose model share falls are those for whom the strip and the tie-break outweigh the far-side gain—one loses 5.9 points to the tray alone—and the five whose share rises are those for whom the gain outweighs both, up to +32.0 points. The copy has no such competition: every participant gains and none loses, because in this layout neither loss term can arise—the box stands on the mat, and its projected region is clear of the others. S7 gives the three components for each participant and object, and S9 gives, participant by participant, the viewpoint geometry, the coverage, and the shares that this section reports only as medians and ranges. The same spread appears in every row of Table 2 that moves: for the copy, the height contribution is +0.1 in one and +10.3 in another, and for the unassigned share, +0.9 in one and −42.5 in another. Read as sign tests over the ten participants (Table 2b), the copy’s height contribution and every margin contribution are consistent in direction (10/10, *p* = 0.002), and the unassigned share falls in nine of ten under height (*p* = 0.021). The model’s height contribution is not consistent (5/10, *p* = 1.0). The bootstrap interval for the model’s median, [−1.8, +11.0] points, spans zero; the copy’s, [+0.3, +7.1], does not (S7). The group median is therefore not a safe summary of what a change of region does to a given participant’s numbers, and the per-participant values (Figure 3 and S9) are the result, with the medians as their summary.

Analysing the two imitation blocks separately changes none of this (S10a). The copy’s height contribution is positive in every participant in the first block and in nine of ten in the second, where one participant is unchanged; the model splits five against five in both; the margin contributions to the model, the copy, and the unassigned share keep their sign in all ten participants in both blocks, and the trays’ is positive in all ten in the first block and in eight in the second, with the other two remaining unchanged; and the medians differ from the pooled values by less than three points. The blocks are not identical in behaviour: the second was shorter in eight of ten participants, and under flat + 19 mm nine of ten spent a larger share of their gaze on the trays in it. Something therefore changed between the blocks, whether through practice, fatigue, or the layer order of the model, and this comparison cannot separate these. The group summaries of what the choice of region did to the numbers did not change; within a participant, a contribution can differ between the blocks by up to 12 points (the margin’s contribution to the model is 26.6 in the first block and 14.2 in the second for one participant, and the model’s height contribution is −10.1 and +0.1 for another; T28), which is one more reason to report the values per participant.

The main contrasts were also repeated on the parts of the blocks where almost every fixation reached the surface, and with the participants divided by coverage (S10f). On the first two thirds of each block, which hold 50 of the 453 fixations that did not reach it and where every participant’s coverage is at least 94.3%, the margin’s contribution is still the larger one at the group median (copy +5.9 against +0.4 points, unassigned −13.2 against −2.3), as it keeps its sign in all ten participants for the model, the copy, and the unassigned share, and the model’s height contribution still splits five against five; the copy’s height contribution is positive in eight participants and zero in two, and smaller than over whole blocks, because much of it comes in the last third (median +4.7 points), when the copy is close to the height of the proxy. Divided by their coverage over the blocks, the five participants whose fixations reached the surface least often (73.3% to 95.3%) and the five who reached it most often (95.6% to 100%) both show a larger margin contribution and the copy’s rise in every participant, but the model does not divide the same way in the two: one rises, and four fall in the first group and four rise and one falls in the second, although the largest rise, +32.0 points, is in the first. The first group is the five participants who account for 407 of the 453 fixations that did not reach the surface. With five participants in a group, this is a description, not a test.

Recovering the viewpoint per frame rather than once per participant changes little where the two can be compared: on the 2498 fixations with an accepted pose, and at the same 19 mm margin, each region’s dwell share moves by a group median of 0.0 points and by at most 2.5 points in any one participant and region (S2). That subset is thin for some participants—52 to 450 fixations, 10% to 98% of each participant’s analysed fixations (S10c)—so this is a check that the fixed viewpoint is not misleading where a per-frame one exists, not a demonstration that the two agree on the whole analysed set. The threshold at which a pose is accepted also does not decide the picture: on the per-frame variant, over the accepted-pose subsets that twelve combinations of the pose tolerance and the reprojection-residual threshold define, the unassigned share stays between 21.5% and 31.7% under the bare footprint and between 1.0% and 1.6% under the raised region with the margin, and only the tightest pair leaves a participant with too few accepted poses to enter the comparison at all (S5). Those figures are for the subsets, not for the 4798 fixations of the main analysis.

Widening the margin buys little assignment but adds overlap. Varying the margin does not change the direction of the height contribution: over the 14 to 24 mm range of Section 2.2, the copy’s contribution stays between +1.7 and +2.9 points and is positive in all ten participants; the model’s median stays near zero, between −0.2 and +1.5 points, with the participants divided five against five throughout, and the unassigned share under the flat region with the margin falls from 10.0% to 6.5%. Within that range, the direction and the rough size of every measure reported here are unchanged; the whole sweep is in Supplementary Material S1b. What the margin does change is something else. Figure 4 takes the raised regions and widens them from the 19 mm used here to 40 mm, on the same fixations and the same viewpoints (Section 2.3). The share of fixation time assigned to some object’s area is already high at 19 mm, at a participant median of 98.3%; widening to 40 mm raises it by a within-participant median of +0.81 points [0.00, 3.19], to a participant median of 99.7%, and two of the ten participants gain nothing at all. Over the same range, the share of fixation time that qualifies for more than one region, counted before the overlap-resolution rule is applied, rises from a participant median of 0.0% at 19 mm to a participant median of 13.1% at 40 mm [0.1, 42.4], passing 1% at a 28 mm margin. The 0.0% at 19 mm is a median, not an absence: four of the ten participants already have some, up to 5.14%, and by 35 mm every participant does.

The two curves are worth reading together before a margin is chosen. This is not a procedure for choosing one: a value read off curves like these depends on the criterion and on the range searched. For the fixations that qualify for more than one region, region membership alone does not determine a unique label, and the overlap-resolution rule determines the final assignment; every fixation still receives one label, so the overlap does not appear in the dwell shares of Table 2.

### 3.2. The Raised Region Agrees with the Raters’ Area of Interest More Often, and Also Assigns an Object’s Area More Often Where They Named the Mat

Table 3 gives the check of Section 2.3 for both raters. Two things follow from it, and they point in opposite directions.

Where a rater put the marker on an object or on its support surface, the raised region with the margin assigned the corresponding area of interest more often than the bare printed region did: 115 of 117 items against 93 for the first rater, and 121 of 124 against 100 for the second. Most of that difference comes from the margin, which already reaches 107 and 113. The two raters agreed with each other on 98.2% of the 167 items usable for both; Cohen’s κ = 0.975 [36]; with the covered items kept, on 97.4% of 196, κ = 0.966. That agreement is high because the task set to the raters is coarse: four working areas, tens of millimetres apart on the mat, judged from a crop centred on the marker, with the items either the rater found covered or unclear removed beforehand; a finer, object-level coding would be expected to agree less. It is the reproducibility of reading where the marker sits against that category set, and it is not evidence that the marker sits where the participant was looking (Section 4.2).

Where a rater put the marker on the mat surface, the raised region assigned an object’s area anyway in 49 of 54 items for the first rater and 47 of 52 for the second, against 28 and 25 under the bare footprint. Rather, more than half of those assignments fall inside a printed region and would have been made without any margin or height, but 9 of the 49 lie more than 19 mm outside every printed region, beyond the margin, and are reached only because the region was given height; the counts by distance for both raters are in S4. That a rater should call the mat while the mapped point sits inside a printed region is not a contradiction: the printed regions are working areas, and they are larger than the objects standing on them. Of the 28 such items for the first rater, 15 fall in the model’s region—printed at the size of the turntable base, 84.4 by 82.5 mm, around a model of 6 by 4 studs—10 in a tray region of 154 by 230 mm, and 3 in the copy’s region. Counted the other way, the raised region assigned a fixation to a different area from the one the rater’s label was folded onto in only 2 of the 171 items. The bare footprint made fewer such cross-area assignments but many more non-assignments: of the 37 items the first rater labelled as the model, it left 13 unassigned. The full confusion of the raters’ seven labels against each rule is in S8.

Of the 200 items, the raised region assigned 195 to an object’s area, before any exclusion. Within those 195, the first rater flagged the marker as covered by a hand or by a held brick in 13.8% of cases and labelled the turntable or the board beneath the model in 10.8% and a bare part of the copy’s baseplate in 6.7%; for the second rater, it was 11.3%, 10.8% and 7.7%. The last two are inside the folded areas of interest and so count as agreement above; they are the part of that agreement that the check cannot attribute to the object itself. The coverage flag is independent of the label, so an item can enter more than one of the three figures.

### 3.3. Transitions, Disagreements, and Where the Projected Regions Overlap

Transition rates move in the same direction as the dwell shares and by a comparably modest amount once the margin is held fixed: model to copy runs at 13.61 per minute under the flat region with a margin and 16.17 under the raised one, and a within-participant difference of +2.58 [−0.53, +12.25] with eight of ten on the same side; tray to copy runs at 3.54 and 4.60, a difference of +0.88 [+0.00, +2.20] with nine of ten on the same side. These figures do not depend on the 1 s gap of Section 2.3: the counts are identical with the limit at 0.5 s, at 2 s, and with no limit (S10b).

Most of the disagreement between the two regions is gaze that one assigns and the other does not. A small part is not: 48 fixations—1.2% of the 4139 that the flat region with a margin assigns to any area, in five participants—are assigned to the model’s area by the flat region and to the right tray’s area by the raised one. We report this to show that the two regions are not related by one simply claiming a superset of the other’s fixations; we cannot say which label is correct for those fixations, since nothing in these recordings determines that. All of them involve the same two objects, whose printed regions are 40.3 mm apart, which is the smallest separation on the mat. These 48 are fixations that the two rules label differently; they are not the same count as the 37 fixations that fall inside the intersection of the two projected regions, reported next, which is a property of the layout rather than of the labels.

Those two objects are also the only pair whose regions overlap once the regions carry height. Carried to the mat plane (Figure 2c), the model’s and the right tray’s regions intersect at every one of the ten viewpoints, by 597 to 880 mm^2^ (median 698), and 37 of the analysed fixations fall in that intersection. No other pair intersects, and the flat regions do not intersect at all, with or without the margin. The overlapping pair does not involve the copy, so none of this rests on the height taken for it.

Both the overlap and the move that clears it grow with the margin. Over the 14 to 24 mm range of Section 2.2, the same pair overlaps at every margin, from 212 mm^2^ at a 14 mm margin to 1878 mm^2^ at a 24 mm margin at the worst viewpoint, and the smallest translation of the model that clears it at every viewpoint rises from 10 mm to 30 mm over the same range. At the 19 mm margin used here, moving the model 20 mm away from the participant, or the right tray 15 mm towards the participant, removes the overlap at all ten viewpoints. These are the smallest single-object moves on that grid, by total displacement in x and y; we did not search moves of both objects at once, and they are not a continuous optimum. They are computed with the viewpoints fixed at the ones we observed, one per participant; a participant who sits elsewhere, or who moves during a block, changes both the overlap and the move that clears it, so these are estimates for this set of viewpoints and not constants of the layout, and moving an object may also move the participant. The search conditions and the results under each height and each margin are in Supplementary Material S6, with the code and tables.

## 4. Discussion

### 4.1. What This Means for a Tabletop Study

A study that draws a region on a printed footprint and adds nothing is making two choices at once: how large the region is and whether it carries the object’s height. These recordings allow the two to be separated. Under the bare printed footprint, a fifth of the analysed gaze was assigned to no area at all, and a margin the size of the tracker’s measured error removed most of that. At the group median, in this layout and at this margin, the margin was the larger of the two contributions. That ranking is a property of these conditions, not a rule: it depends on the objects’ heights and distances, on the layout, on the margin and on the tracker’s accuracy, and a taller or closer object, a narrower margin or a more accurate tracker would shift the balance towards height.

The group median is not the whole result. At the same margin, the height of the region moved a participant’s copy share by anything from 0.1 to 10.3 points, and the model’s from −5.7 to +32.0, with the participants divided five against five (Section 3.1). The direction was the same in all ten participants for the copy, but direction alone says nothing about size.

We draw four recommendations.

First, report what the choice of region does to each participant, not only to the group. The effect of the rule is a per-participant quantity, and a single group figure, in either direction, will misdescribe some of the sample: on the model, the group median is +0.4 points, while two participants lie at −5.7 and +32.0, and on the copy, every participant increases but by between 0.1 and 10.3 points. A study reporting only group medians under one region cannot say how much its own participants were affected.

Second, where objects stand tall and close together, lay the objects out so that the projected regions do not overlap, and check this before recording. In this layout they do overlap (Section 3.3). The ambiguity is created by giving the regions height, on a layout that was drawn without height in mind. Piloting is where a check of this kind belongs, and the practice is established: the worked example in the current piloting guidance is a marker-covered table on which two participants build a brick model together [37]. What that guidance offers for areas of interest is how many to define and which of them to keep; whether two of them overlap once they carry height is not visible in a pilot recording, and has to be computed from the footprints, the heights and the viewpoints.

What has to be cleared can be stated precisely, for a given set of viewpoints. The printed regions plus their margins leave 2.3 mm between the model and the right tray. The projection then moves the tray’s far edge away from the participant by 32 to 45 mm and the model’s near edge (the top of the turntable base, at 16.0 mm) by 15 to 21 mm, so the clearance required is the difference between the two, 17 to 24 mm across the ten viewpoints, against the 2.3 mm available. The quantity to be cleared is the difference between the displacements of the two edges that face each other, not the displacement of either object on its own. That one-dimensional estimate is conservative here by about 4 mm against the exact polygons, so it is usable for choosing a layout, and the polygons are worth computing before fixing one. All of these distances are computed at the ten viewpoints we observed, held fixed within each participant; they are estimates to be recomputed for another layout and the viewpoints its participants will take, and a head that moves during a block moves them too.

Of the four recommendations, the first, third and fourth do not depend on the task or the layout: what a region does to each participant, what was assigned to nothing, and what a wider region costs are quantities any tabletop study can report. The second is the one whose content changes with the setting—the pair of objects that overlaps, and by how much, is a fact about this layout and these viewpoints—and what carries over is the check, not its result. A reader can run the same check before recording, in this order: measure each object’s footprint and height range; fix the viewpoint, or the range of viewpoints, the participants will use; expand each footprint by the margin; raise it to the object’s height; project it onto the table to get its planar region; and then test the regions pairwise for overlap and move the objects until none remains. Clearing the overlaps settles only which object a region can claim. It does not reduce unassigned gaze, occlusion, objects that move, or the tracker’s error.

Third, report the region’s extent and margin, and report the unassigned share. The same gaze inside the same analysed surface was 21.6% unassigned under one defensible region and 1.7% under another. A reader said only the first figure has no way to separate what the rule contributed from what the participants or the tracker did. The same quantity separates an automatic rule from a human coder: in a real-world body-directed task, an algorithm left 35.9% of frames outside any region while human raters left 13.9%, a difference the authors attribute to raters applying a tolerance when gaze fell slightly outside a region [29]. There is at present no expectation that this be reported: the consensus minimum reporting guideline for eye tracking covers the tracker, the calibration, the measurement uncertainty and the data loss, and states that it does not repeat items belonging to intersecting fields; areas of interest are not among its items [38]. How a region was drawn is therefore outside what a study is currently asked to report, while on these recordings it moved the unassigned share from 21.6% to 1.7% at the group median.

Fourth, do not read a higher assignment rate as higher accuracy, and check what a wider margin costs before widening one. The choice of region is a trade-off between coverage and precision: a wider or taller region assigns more of the gaze, and it also admits more gaze that does not belong to the object, so a region acts as a classifier that can either miss gaze that belongs to the object or admit gaze that does not, and shrinking one of those errors enlarges the other, which is how areas of interest have been framed for flat stimuli [8]. Both the margin and the height claim gaze that the smaller region did not, and neither step has been shown here to claim the right gaze, only to claim more of it; Figure 4 quantifies the coverage side, and the check of Section 3.2 bounds the other side only as far as a rater’s reading can. On these recordings, the assigned share was already at a participant median of 98.3% at the margin used, and widening it to 40 mm added a within-participant median of 0.81 points while taking the fixation time that qualifies for more than one region from a participant median of 0.0% to 13.1% (Figure 4). The caution is not ours: the study quoted in Section 1, which raised the share of interpretable fixations by widening the assignment criterion, states it in the same place [24]. The trade-off itself has been quantified before, on gaze simulated towards a face with the target known: shrinking the region reduced the share assigned to a neighbouring feature, but left up to 43% of fixations in no region at all [25]; Figure 4 is the counterpart of that result on physical objects, measured rather than simulated, with the footprints and the tie-breaking rule held fixed. The second quantity is worth computing alongside the first, on one’s own layout and viewpoints.

### 4.2. Limits

The raised region is not an independent standard. It recovers gaze that the flat region loses, and the direction of the flat region’s loss follows from the projection. But a raised region also claims everything on the segment between the viewpoint and the region, including gaze directed at a hand or at a brick in transit, and any mapped point beyond the object’s footprint, bare mat included.

A raised region cannot be separated from a systematic gaze offset. The region grows away from the viewpoint by D·h/(C_z − h), so it is an anisotropic margin pointing in one direction, and it absorbs any error that points the same way. The participant with the largest height contribution on the model, +32.0 points, is also the one seated furthest back (C_y = 704 mm against 577 to 668 mm for the others) and the one whose gaze lands furthest beyond the model: the median mapped position near the model is y = 106 mm for that participant against 131 to 198 mm for the rest, and the share of their fixations that reached the mat surface at all is the lowest in the sample, 73.3% against 81.9% to 100%. Their projected displacement, 107 mm by the same construction as Table 1 (S9), is not the largest—another participant’s is 118 mm with a height contribution of only +5.3 points—so geometry alone does not account for the difference. The offset correction recorded for the session also cannot settle it: this participant received one of 1.0°, while the two largest corrections, 4.1° and 6.2°, went to participants whose model contribution was −1.8 and −0.5 points (S10e). A correction records how far the gaze was shifted at the start of the session, not how much error remained afterwards; a small one does not show that a participant’s gaze was accurate, nor a large one that it was not, so the corrections can neither explain this participant’s difference from the others nor rule out residual error as its source. Whether the raised region recovered gaze that belonged to the model or absorbed an offset that happened to point the same way cannot be decided from these recordings. This is the same limitation as the margin’s: it was measured once, on one wearer, and it is not a per-participant figure; the corrections of Section 2.3 say that six participants’ gaze was visibly displaced at the start of the session and by how much it was shifted, not how accurate it was afterwards. Assigning each fixation to a pre-defined category by hand, under the gaze marker, is the alternative that needs no region at all [28]. The check of Section 3.2 applies that alternative to a sample of 200 fixations, and what it can and cannot settle is worth stating plainly. An independent record is a different thing again: integrating gaze with motion capture expresses the point of gaze in the laboratory frame and allows it to be compared with independently measured target positions, which gives a mean accuracy of 2.55° that degrades with viewing distance [17], and the same integration is what yields gaze vectors in three dimensions [18]. Both bypass the marker plane rather than testing it, and we are not aware of a study that validates plane-mapped gaze against a record of that kind.

An area of interest is a working area, not an object. The agreement in Table 3 is with the folded categories of Section 2.3, in which the turntable counts as the model’s area and the bare baseplate as the copy’s area. The two rules do not treat the turntable alike: the raised region begins at its top surface, 16.0 mm above the mat, so a fixation on the turntable’s own side is outside the box, while a rater who named the turntable is counted as naming the model. Extending the box’s floor down to the mat, which removes that asymmetry, moves the model’s share by a group median of 0.2 points and by at most 2.4 points in any participant—computed on the main analysis itself, at the fixed viewpoint and on all 4798 fixations (S7)—so the disagreement is small here; but it is a choice, and a study drawing an object-level conclusion has to make it explicit rather than inherit it from how the footprint happened to be printed. The areas of interest are therefore defined as working areas that include the surfaces that the objects stand on, which is what the printed footprints are. The check consequently cannot separate looking at an object from looking at what it stands on: by the counts in Section 3.2, roughly one assignment in six is inside the right working area without being on the object itself. A study drawing an object-level conclusion, rather than an area-level one, has to say which of the two it means; the choice belongs with the region-definition decisions that are catalogued among the threats to the validity of eye-movement research [10], and whose consequences are established for flat stimuli [8,9].

The covered items are set aside, and that limits the scope. Where the marker sits on a hand or on a brick the hand is holding, the rater has to separate the object the marker overlaps from the object behind it, and the category the item belongs to becomes a matter of interpretation rather than of what is visible. We therefore take the uncovered items as the primary comparison and report the figures, including the covered ones in S4, where they change the counts little. The exclusions remove 29 items for the first rater and 24 for the second, and 33 from the set on which the two raters are compared. The counts reported above therefore do not generalise to fixations whose marker was covered.

What the check does not establish. The gaze marker on those frames comes from the same eye-tracking estimate as the analysis, so a rater can say what the marker was over, not where the participant was looking; that is a different quantity from an independent record of the gaze target, and agreement between two raters is the reproducibility of a judgement rather than its correctness. Two coders of mobile eye-tracking video have been reported to agree at κ = 0.99 on areas of interest drawn around faces [26], and at κ = 0.80 on fixation locations classified from still frames during walking [27]; the present figure sits inside the range that this kind of coding produces and is not by itself informative about the mapping.

Regions are fixed to printed positions. Participants rotate the turntable and lift the copy off the baseplate. For that part of the data, no fixed region is right, under any of the three definitions, and the unassigned share under the bare footprint runs from 11% to 67% between participants. We did not code when objects were moved, so we cannot say how much of that spread comes from movement rather than from gaze error or from where each participant worked. Handling it requires an independent record of where the objects are, which is what the alternatives provide: a geometric proxy registered to the scene camera [19], a photogrammetric model of the room [20], segmented object masks [21], or markers on the objects and the head [18]. Each buys the moving object at the cost of extra hardware or modelling, and the plane-mapped and three-dimensional families have been set side by side without being reconciled [22]. An intermediate option keeps the plane and lets the region move on it: an area of interest that follows the object from frame to frame, allocated on the video manually or semi-automatically, as Faraji and colleagues do for moving objects in videos shown on a wide screen [4]. That handles the object that is moved and the copy that grows, at a cost in coding, but it does not remove the displacement studied here: a region that follows the object on the plane still meets the gaze ray where the ray crosses the plane, not where it meets the object, so the object’s height has to be added to the moving region in the same way as to the fixed one. The plane can also be bypassed by deciding frame by frame, with a classifier trained on labelled frames, whether the gaze marker in the scene image lies on the moving object [39]; like the check of Section 3.2, it judges where the marker lies, not where the participant was looking.

Scope. This is a methodological demonstration, not a validation of raised regions. Ten participants, all young adult students and all right-handed, one analysed task, one mat, one device, one margin, one session each, twenty blocks and 4798 fixations. The imitation blocks were the first two blocks of every session, so the results describe that task in that position: an order effect relative to the free-design task was not tested, and the block-by-block analysis of S10a covers only the opening minutes of a session. What the recordings establish is what two definitions of a region do to the same data; the sizes reported—the contributions in Table 2, the overlap areas and the moves that clear them—belong to these objects, heights, distances and viewpoints and this margin, and only the pattern can be expected to carry over: that size and height are separable choices, that the copy’s gain is fixed in direction by its geometry, and that the model’s competing gain and loss can split a sample. Whether raised regions are valid in general, and how large the two contributions are elsewhere, is a matter for replication with other layouts, objects, tasks, trackers and participant groups, with gaze accuracy measured per participant. Sex and gender are not reported. The ten participants were recruited from a small pool at the authors’ own institutions, and any breakdown would risk identifying individuals to those who know that pool; every contrast is within-participant, and no group is compared with another, so the design supports no stratification by that or any other participant attribute. The turntable, the model, and the trays were measured, but the copy’s height changes as it is built and was not measured: the finished model’s height was applied to it throughout, so the height contribution reported for the copy rests on that proxy. Repeating the main analysis with only that height changed, on the same fixations, viewpoints and margin, gives a copy contribution of +1.7 points at 24 mm, +2.4 at 52.5 mm and +2.6 at 96 mm, positive in all ten participants at each (S3b). The summary contribution of height for the model and the trays is the same at every copy height, +0.4 and +0.1 points, but the assignment is not quite unchanged: at 96 mm the copy’s region takes a few fixations that the model otherwise holds, and the model’s group-median share moves from 53.1 to 53.0. The margin rests on a gaze error measured once for this setup, from one wearer in one separate session, and is not a per-participant figure (S1); how such a figure is to be determined and reported is itself the subject of standing guidance [16]; Supplementary Material S1b shows what happens to the height contribution when it is varied from 14 to 24 mm. A study whose tracker is less accurate would need a larger margin, and the balance between the margin and height would shift accordingly. The viewpoint is the scene camera’s optical centre rather than the eye. That is the consistent choice here: the enrichment’s mapped point is where the camera’s ray meets the mat, so the projection that undoes it starts from the same centre; the eye’s own offset from that centre is a separate error, and displacing the viewpoint by 40 mm changes the shares by at most 2.9 points, a figure obtained at the 19 mm margin on the accepted-pose subset of S2 with the per-frame viewpoint rather than on the main analysis (S3c).

## 5. Conclusions

On these recordings, a region’s size and its treatment of height are separable choices, and they have to be reported separately. What follows is what those choices did to ten participants in one layout, with one tracker and one margin, and is offered as a methodological case study of that kind. Holding the printed footprint and the margin fixed, giving the region height increased the dwell share of the copy’s area in all ten participants, by a median of 2.4 percentage points (range 0.1 to 10.3). The unassigned share decreased in nine participants, with a median change of −4.8 percentage points. For the model’s area, the group median was +0.4 points, with five participants increasing and five decreasing and extremes of −5.7 and +32.0; the group median therefore did not capture the opposing changes across participants. The effect of the rule is a per-participant quantity, and a single group figure, in either direction, will misdescribe some of the sample. The margin’s contribution, whose importance is already established for flat stimuli, was the larger of the two at the group median under the conditions tested here—this layout, the 19 mm margin, these heights and distances: it raised the copy area’s dwell share in every participant by a within-participant median of 7.1 points and cut the unassigned share in every participant by 14.5 points. The ranking is not a general one, and with other objects, margins, or trackers it may reverse. The two imitation blocks give the same pattern separately, and the fixed viewpoint agrees with a per-frame one to within 2.5 points on the subset where the two can be compared, a subset that is thin for some participants. On the parts of the blocks where nearly every fixation reached the surface, the margin’s contribution is again larger, and the model’s height contribution again splits five against five, while the copy’s gain from height is smaller and absent in two participants; divided by coverage, the participants with lower and higher coverage do not split the same way on the model. Every share reported here is a share of the fixation time that reached the localized surface.

The four recommendations of Section 4.1 follow: report what the choice of region does to each participant, not only to the group; lay out tall neighbouring objects so that their projected regions do not overlap, and run the check of Section 4.1 before recording; report the region’s extent, its margin and the unassigned share; and treat a higher assignment rate as a change in bookkeeping rather than as evidence of accuracy. Where objects stand tall and close together, the two rules can place the same fixation in different objects’ areas, and computing both is cheap enough to be worth doing before an object-level conclusion is drawn. The areas compared are working areas, each an object with the carrier it stands on, so what these recordings establish is assignment to an area, not gaze verified on the object itself. Neither region was checked against an independent record of what was looked at, so their relative accuracy remains undetermined; the video check compares the rules with what a rater could see under the gaze marker, and there the raised region both agreed with the rater’s area of interest more often and assigned an object’s area more often where the rater saw only the mat. Coverage and validity are therefore two quantities, and a study that adopts a raised region needs to evaluate both how much of its gaze the region claims, which these recordings measure, and how much of what it claims belongs to the object, which they only bound.

## Figures and Tables

**Table 1:** The objects: printed region, the height used, the viewing geometry, and the displacement of the plane crossing that follows. It is computed from geometry alone, using the fixed per-participant viewpoint of Section 2.2. Every quantity with a range is one value per participant, and the Table gives the median over the ten participants with the minimum and maximum in parentheses. Height is the range that the object occupies above the mat, and h in the displacement is its top. Horizontal distance D is measured from the viewpoint to the centre of the printed region; the line of sight is measured from the viewpoint to the centre of the object’s top surface; the viewpoint height C_z is 417 mm (range 364 to 441) across the ten participants. The displacement is D·h/(C_z − h): the distance by which the point where the line of sight to the object’s top meets the mat plane lies beyond the printed footprint, away from the participant. The last column converts an angular error of 1.45°, the mean inaccuracy reported for this tracker with the same surface mapping (Section 2.2), into a distance along the line of sight to that object; it is the conversion behind the range quoted in Section 2.2. No fixation enters this Table.

Object	Printed Region (mm)	Height Above the mat (mm)	Horizontal Distance D, Median (min–max) (mm)	Line of Sight to the Top, Median (min–max) (mm)	Displacement D·h/(C_z − h), Median (min–max) (mm)	1.45° at That Line of Sight (mm)
Model (on turntable)	84.4 × 82.5	16.0 to 68.5	484 (424–540)	594 (516–643)	96 (84–118)	15.0
Copy (on baseplate)	63.8 × 63.8	0.0 to 52.5	273 (208–334)	458 (374–494)	39 (33–49)	11.6
Tray, left	154.0 × 229.6	0.0 to 30.0	330 (286–402)	513 (440–557)	26 (23–31)	13.0
Tray, right	154.0 × 229.6	0.0 to 30.0	354 (312–393)	522 (457–551)	28 (24–35)	13.2

**Table 2:** (a) Dwell-time share of each object’s area (Section 2.2) and the share assigned to no area, as group medians under each region; every share is of the fixation time that reached the localized surface (Section 2.3). (b) The within-participant contribution of height and of the margin: height alone is raised + 19 mm minus flat + 19 mm and margin alone is flat + 19 mm minus flat, each computed within each participant and summarised as the median with the minimum and maximum in brackets. “Up/down” counts the participants whose value rose and fell (the ten minus both is the number unchanged); for the no-area row, a fall is a decrease in unassigned gaze. The sign test is two-sided over the participants on either side of zero (S7). The tray row pools the areas of the left and right trays, which Table 1 lists separately. The two margin maxima that print as +20.7 belong to different participants (20.67 for the model area, 20.71 for the copy area before rounding).

**(a) Dwell-time share under each region, group median over the ten participants (%)**						
**Area**	**Flat**		**Flat + 19 mm**		**Raised + 19 mm**	
Model area	41.9		50.5		53.1	
Copy area	19.3		26.3		30.2	
Tray areas	14.1		15.3		15.7	
No area	21.6		8.1		1.7	
**(b) Within-participant contribution of height and of the margin (percentage points of dwell-time share)**						
**Area**	**Height Alone: Median [min, max]**	**Up/Down**	**Sign Test *p***	**Margin Alone: Median [min, max]**	**Up/Down**	**Sign Test *p***
Model area	+0.4 [−5.7, +32.0]	5/5	1.000	+10.1 [+1.2, +20.7]	10/0	0.002
Copy area	+2.4 [+0.1, +10.3]	10/0	0.002	+7.1 [+1.1, +20.7]	10/0	0.002
Tray areas	+0.1 [+0.0, +6.0]	6/0	0.031	+1.0 [+0.0, +3.7]	10/0	0.002
No area	−4.8 [−42.5, +0.9]	1/9	0.021	−14.5 [−34.2, −7.2]	0/10	0.002

**Table 3:** The check of Section 2.3: 200 fixations were drawn from the analysed set and judged independently by two raters who had seen no assignment, and their seven labels were folded onto the four areas of interest. The raters agreed with each other on 98.2% of the 167 items usable for both (Cohen’s κ = 0.975; Section 3.2), which is the reproducibility of their reading and not a measure of the mapping’s accuracy. Each rater’s own covered and unclear items are excluded from that rater’s column. “Same area of interest” counts only the items where that rater named an object or its support surface; “assigned an object’s area” counts only the items where that rater named the mat surface. S4 gives the same counts with the covered items kept, and under the other ways of folding the labels.

	Rater 1	Rater 2
Items compared, with the rater’s covered and unclear items excluded	171	176
The rater named an object or its support surface	117	124
The rater named the mat surface	54	52
Same area of interest as the rater: Flat	93	100
Same area of interest as the rater: Flat + 19 mm	107	113
Same area of interest as the rater: Raised + 19 mm	115	121
Assigned an object’s area where the rater named the mat: Flat	28	25
Assigned an object’s area where the rater named the mat: Flat + 19 mm	41	39
Assigned an object’s area where the rater named the mat: Raised + 19 mm	49	47

## Data Availability

The original contributions presented in this study are included in the article and the Supplementary Material. The analysis code, the task-window table, the per-fixation table with the assignment under every region and the pose used, the fixed viewpoint per participant, the measured object heights, the gaze-error session behind the margin, the two raters’ judgements and the result Tables are openly available as a single archive at the Open Science Framework, https://doi.org/10.17605/OSF.IO/N5P4S, under CC BY 4.0; the same archive is provided in the Supplementary Material. Running reproduce.sh in that archive rebuilds the derived measures and reproduces every number and Table reported here. The per-frame camera poses are available from the corresponding author on request. Participants are identified by pseudonyms and all timestamps are given relative to the start of each participant’s own session. The raw recordings and the enrichment export from which the per-fixation Table was built are available from the corresponding author on request. Beyond the images published as Figure 1, scene-camera video is not shared, and neither is any further frame taken from it: the terms of participant consent exclude the recorded video from provision or publication. The recordings analysed here belong to a study that is still in progress.

## Supplementary Materials

The following supporting information can be downloaded at: https://www.mdpi.com/article/10.3390/jemr19050114/s1; Supplementary Materials S1–S10 (PDF), an index giving for each item the method, the fixation set and the conditions; Analysis data and code (ZIP), the archive described in the Data Availability Statement, which reproduces every number and table by running reproduce.sh. The items indexed are: S1, the gaze error measurement behind the 19 mm margin, with the intervals used and how the 19.0 mm was obtained; S1b, the whole comparison repeated at every margin from 14 to 24 mm, for all four areas, with the figure that Section 3.1 refers to, and the assignment and overlap shares behind Figure 4 at every margin from 19 to 40 mm; S2, the fixed and per-frame viewpoints compared on the fixations whose pose was accepted; S3, sensitivity to the assumed object heights and to the assumed eye position, computed on the per-frame variant (S3a, S3c), and the height applied to the copy varied on the main analysis itself (S3b); S4, the video check of Section 2.3 and Section 3.2, with the sampling, the two exclusion rules and their denominators, the counts under each region and each of the four ways of folding the labels, the same counts with the covered items kept, and the distance of the mat-surface assignments from the nearest printed region; S5, the assignment under twelve settings of the pose tolerance and reprojection-residual threshold; S6, the layout check of Section 2.2 and Section 3.3, covering the agreement between the planar form of the raised region and the segment-and-box test, the pairwise overlaps of the projected regions at each viewpoint under the three region definitions, the union over viewpoints reported separately, the search conditions, and the results under each copy height and each margin from 14 to 24 mm. S7, the decomposition of the height contribution into what each region gains far from the participant and loses near them, separating the strip left outside the raised box from the projected overlap, with the sensitivity of the model’s share to lowering its box floor to the mat and the sign tests and bootstrap intervals for every contrast; S8, the confusion of the raters’ seven labels against each rule, before the folding of Section 2.3, with the sizes of the printed working areas that explain why a rater could name the mat inside one; S9, one row per participant giving the viewpoint geometry, the coverage, the gaze offset correction recorded for the session, the dwell shares under each region and the height contribution that the main text reports only as group medians and ranges; S10, the checks added at revision—the two imitation blocks analysed separately, the transition rate under other gap thresholds, the accepted-pose subset of S2 by participant, the fixations that did not reach the surface by participant and by third of the block, the gaze offset correction per participant, and the main contrasts by portion of the block and with the participants divided by surface coverage. The per-fixation table with the assignment under every region, the recovered viewpoints, and the code that produces every number and table are included.
