# Supplementary material for: Effects of Object Height and AOI Margins on Gaze Measures in a Tabletop Task
Source: J Eye Mov Res. 2026 Oct 5;19(5):114. doi: 10.3390/jemr19050114 (PMC13705654; doi:10.3390/jemr19050114)
Supplement: Supplementary file 1 [file jemr-19-00114-s001.zip › Supplementary_Materials_S1-S10.pdf]

Supplementary Materials S1–S10

Effects of Object Height and AOI Margins on Gaze Measures in a Tabletop Task

Fumika Suzuki, Mika Isobe, Kazaru Yaegashi, Yuji Mizoue, Mizuki Nakajima and Yusuke Nagamori

---

Main analysis (for reference): fixation set data/main_inunit/ = the 4,798 fixations inside the localized surface (91.4% of those in the imitation blocks), ten participants. Regions: **flat** (P_m0), **flat + 19 mm** (P_m19), **raised + 19 mm** (F_m19, one fixed viewpoint per participant). The per-frame-pose variant is B_* and is **not** the main analysis.

Index

Each item below gives the method, the fixation set it was computed on, and the files that hold it. The main text cites these identifiers directly.

ID	What it supports
**S1**	The 19 mm margin: the median gaze error measured for this setup
**S1b**	Two things the margin does: (i) the height contribution is not critical to the 19 mm choice — the whole comparison repeated at 14–24 mm; (ii) what a wider margin costs — assignment and overlap at 19–40 mm (Figure 4)
**S2**	How far the fixed and per-frame viewpoints differ, where they can be compared
**S3a**	Sensitivity to the assumed object heights
**S3b**	Sensitivity of the main analysis to the height applied to the copy
**S3c**	Sensitivity to the assumed eye position
**S4**	Video check against two independent raters (Sections 2.3 and 3.2)
**S5**	The per-frame-pose analysis under other settings
**S6**	The layout check of Sections 2.2 and 3.3: that the planar form of the raised region agrees with the segment-and-box test, where the projected regions overlap in the present layout, and what moves remove the overlap
**S7**	The decomposition behind Section 3.1: why the copy moves in one direction in all ten participants while the model splits five against five, and the sign tests for every contrast
**S8**	The confusion behind Section 3.2: what each rule assigned against each of the raters' seven labels, and why a rater could call the mat while the mapped point sat inside a printed region
**S9**	One row per participant, for the quantities the main text reports only as group medians or ranges
**S10**	Six checks added at revision, on the same fixation table and region labels as the main analysis: **(a)** the two blocks separately; **(b)** other transition gap thresholds; **(c)** the accepted-pose subset by participant; **(d)** the excluded fixations by participant and block phase; **(e)** the gaze offset correction per participant; **(f)** the main contrasts by portion of the block and with the participants divided by surface coverage

S1. The 19 mm margin: the median gaze error measured for this setup

Method. One wearer, one session, separate from the ten participants of the main analysis. The wearer pointed in turn at the fixation dots printed on the validation mat; the target coordinates are the nominal printed positions from the mat's design file (data/validation_points.csv, rows with kind = fixation, columns mat_x_mm / mat_y_mm), not positions measured after printing. **Selection** (confident in the source table): the fingertip had to be within 25 mm of the nearest printed dot **and** at least twice as far from the second-nearest, and the window had to hold at least 20 gaze samples flagged as on the surface. **Window**: from 0.5 s after the marked instant to 3.0 s before the next mark, with a 0.2 s floor. **Summary**: the median x and median y of the gaze in that window, in mat coordinates; the error is the distance from that median point to the target's printed coordinates, the precision the median distance of the samples from their own median, and the angular equivalent uses the target-to-camera distance at the window's midpoint. **19.0 mm is the median of the 11 per-target errors, not a median over gaze samples** (the 11 windows hold 7,723 samples). Range 6.8–50.8 mm; median precision 5.6 mm; target distances 581–834 mm. Three other lead/lag settings give medians of 20.6, 21.6 and 21.7 mm

Fixation set. That session only; 11 of the 15 segments in the source table. The four dropped: three had a fingertip 85–129 mm from the nearest dot; one (segment 14) had a fingertip 15.2 mm away and failed the second-nearest condition

Files. tables/TA1_gaze_error_targets.csv (the 11 used), data/gaze_error/T12_gaze_against_pointed_target.csv (all 15 segments with the confident column), data/gaze_error/T13_gaze_window_sensitivity.csv (the lead/lag settings), data/validation_points.csv (the mat's design file: 16 fixation dots, 4 marked gaze_cal and 12 gaze_eval, with their printed coordinates)

S1b. Two things the margin does: (i) the height contribution is not critical to the 19 mm choice — the whole comparison repeated at 14–24 mm; (ii) what a wider margin costs — assignment and overlap at 19–40 mm (Figure 4)

Method. (i) Labels recomputed from the stored normalized positions and the fixed per-participant viewpoint, 1 mm steps, same assignment and tie-breaking rule; the 19 mm condition reproduces the stored `P_m19` and `F_m19` labels exactly (0 mismatches, checked in `tables.py`). (ii) The same raised regions with only the margin widened: the assigned share is the dwell time entering at least one box, the overlap share the dwell time entering two or more, counted **before** tie-breaking. Neither is a rule for selecting a margin; a value read off these curves depends on the criterion and the range searched

Fixation set. The analysed set (4,798 fixations) at every margin

Files. `tables/TA2_margin_sweep.csv` (group medians, 14–24 mm), `tables/TA2b_margin_sweep_by_participant.csv` (one row per margin × participant × object), Figure S1b below (the height contribution at 14–24 mm for all four areas, one line per participant, median in black, 19 mm dashed — the figure Section 3.1 refers to), `tables/TA4_margin_tradeoff.csv` (assigned and overlap share per margin × participant, 19–40 mm; the source of Figure 4)

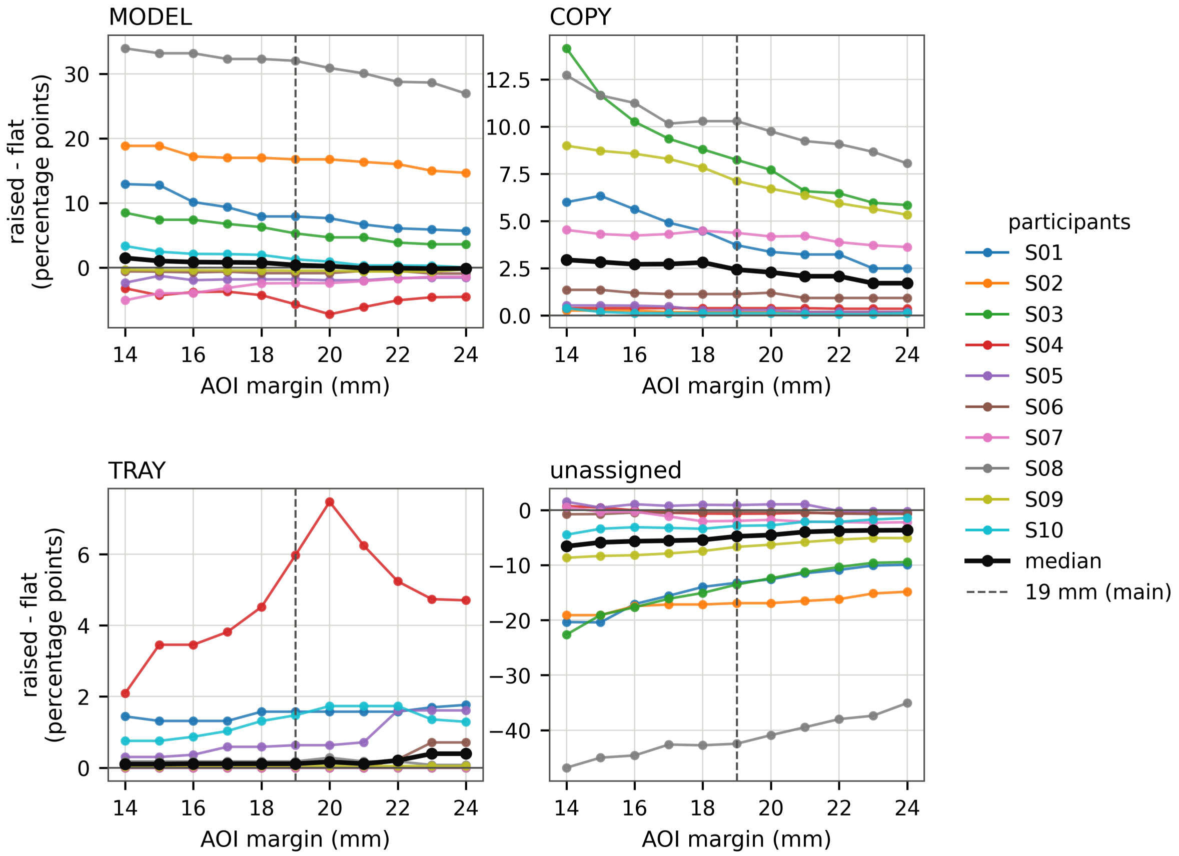

Figure S1b. The contribution of height over areas-of-interest margins from 14 to 24 mm in 1 mm steps, on the same 4,798 fixations and the same per-participant fixed viewpoint as the main analysis. Each panel plots, for one area, the raised region minus the flat region at the same margin, in percentage points of dwell-time share: the model, the copy, the trays, and the share assigned to no area. One coloured line per participant (n = 10); the black line is the median over the ten participants; the dashed vertical line marks the 19 mm margin used in the main analysis and the horizontal line marks zero. Drawn from `tables/TA2b_margin_sweep_by_participant.csv`.

S2. How far the fixed and per-frame viewpoints differ, where they can be compared

Method. `F_m19` (one viewpoint per participant) against `B_m19` (viewpoint per frame), at the same 19 mm margin, both evaluated on the same subset

Fixation set. The 2,498 fixations of the imitation blocks with an accepted pose (time difference ≤ 100 ms, reprojection RMS ≤ 10 px); this is a subset of the 4,798 analysed, so it does not test the main analysis as a whole. **The subset is not evenly spread over participants**: it holds 52 to 450 fixations per participant, 10.4% to 98.3% of that participant's analysed fixations (S10c, `T30`), so the comparison is thin for some participants, and the differences in `T11` describe that subset only

Files. `tables/T11_fixed_vs_dynamic.csv` (group summary), `tables/T30_pose_subset_by_participant.csv` (the subset and the per-frame minus fixed difference per participant)

S3a. Sensitivity to the assumed object heights

Method. Dynamic B_m19 variants (B_m19_turn0/10/30, build24/96, tray0/40). **This is a check on the per-frame method, not on the fixed-viewpoint main analysis.**

Fixation set. Accepted-pose set as in S2

Files. tables/T10_height_sensitivity.csv

S3b. Sensitivity of the main analysis to the height applied to the copy

Method. The main analysis itself: same fixations, same fixed per-participant viewpoint, same 19 mm margin, same assignment and tie-breaking rule, only the copy's height changed (24, 52.5, 96 mm). The 52.5 mm condition reproduces the stored F_m19 labels exactly (0 mismatches). The 96 mm condition is a test of the assumption, not a height the copy reached in the task

Fixation set. The analysed set (4,798 fixations)

Files. tables/TA3_copy_height.csv

S3c. Sensitivity to the assumed eye position

Method. Camera centre displaced ±20/±40 mm on each axis, box assignment repeated

Fixation set. Accepted-pose set as in S2

Files. tables/T14_eye_offset.csv

S4. Video check against two independent raters (Sections 2.3 and 3.2)

Method. Every region (P_m0, P_m19, F_m0, F_m19, and B_m0, B_m19 on the accepted-pose subset) against each rater's labels. Two exclusion rules, used for different quantities: against a region, that rater's own covered and unclear items are dropped (29 items for rater 1, leaving 171; 24 for rater 2, leaving 176); between the two raters, the covered and unclear items of either are dropped (33, leaving 167). The reference figures keep the covered items and drop only the unclear ones (198 for each rater against the regions, 196 between the raters). The seven labels are folded onto the four areas of interest in four ways, PLATE→copy or unassigned × BASE→model or unassigned; the main text uses copy and model, and all four are tabulated

Fixation set. 200 fixations drawn from the analysed set: 20 per participant, stratified by block and by the first/middle/last third of the block, fixed seed; region labels not used in the sampling

Files. tables/T18_interrater.csv (agreement, κ, and what each region assigned, over all 8 folding × exclusion settings), tables/T19_videocheck_detail.csv (the counts of Table 3, and the distance of the mat-surface assignments from the nearest printed region), video_check/ (the two raters' tables, the written instructions they were given, and the selection key; these are human judgements and nothing in the archive regenerates them).

S5. The per-frame-pose analysis under other settings

Method. B_* under 4 pose tolerances × 3 residual thresholds. Over the twelve accepted-pose subsets (2,163 to 3,763 fixations) the unassigned share runs 21.5–31.7% under the bare footprint and 1.0–1.6% under B_m19; these are subset figures, not the main analysis

Fixation set. data/sens/tol*_rms*/, rebuilt by reproduce.sh (not shipped)

Files. tables/T13_sensitivity_grid.csv

S6. The layout check of Sections 2.2 and 3.3: that the planar form of the raised region agrees with the segment-and-box test, where the projected regions overlap in the present layout, and what moves remove the overlap

Method. Central projection of each raised, margin-enlarged region onto the mat plane, from the fixed per-participant viewpoint; pairwise polygon intersection; grid search over translations

Fixation set. All 4,798 analysed fixations for the agreement check; geometry only for the overlaps and the search

Files. layout_design/out/step1_summary.json (agreement; the per-fixation file step1_verification.csv is regenerated by reproduce.sh but not shipped), step2_* (overlaps by viewpoint and the union over viewpoints, reported separately), step3_* (search conditions and solutions; the per-candidate file step3_single_move.csv is regenerated but not shipped; single-object moves only, minimised by the sum of the absolute translations on a 5 mm grid over ±80 mm — moves of both objects at once were not searched; results under the copy heights examined, 24 and 52.5 mm, and under each margin from 14 to 24 mm in step3_margin_sweep.csv), step4_clearance.csv (the clearance estimate)

S7. The decomposition behind Section 3.1: why the copy moves in one direction in all ten participants while the model splits five against five, and the sign tests for every contrast

Method. For each participant the difference between `F_m19` and `P_m19` is split into three parts by dwell time: **gain_far**, the time the raised region claims that the flat region left unassigned; **lost_near_tie**, the time the flat region assigned to that object's area and the raised region gave to another under the tie-breaking rule; **lost_near_none**, the same but left unassigned. The copy's box stands on the mat, so its raised region contains its flat one; and in the present layout at the 19 mm margin its projected region overlaps no other object's (S6). Both conditions are needed for the loss terms to be zero: the first rules out a loss to unassigned, the second a loss to another object's area under the tie-breaking rule. Here both hold, so both terms are zero. The model's box begins at the turntable's top surface (16.0 mm), so a strip of its flat region nearest the participant falls outside the box. The losses are classified by what the line of sight actually enters: no box at all (unassigned), only another object's box (still the strip), or both boxes with the tie-break awarding the other (the projected overlap of Section 3.3). S7 also carries the sensitivity of the model's share to lowering the box's floor to the mat, computed on the main analysis at the fixed viewpoint over all 4,798 fixations. Sign tests are two-sided over the ten participants; the confidence intervals are percentile bootstrap of the median, 20,000 resamples, fixed seed. **These tests are reported for completeness; nothing in the main text rests on a threshold**

Fixation set. The analysed set (4,798 fixations)

Files. `tables/T20_height_decomposition.csv` (one row per participant × object), `tables/T21_gain_loss_position.csv` (how far from the participant the gained and lost fixations were mapped, and how many of the losses went to another object's area), `tables/T22_sign_tests.csv`, `tables/T24_model_loss_kind.csv` (each lost fixation classified as strip or tie-break), `tables/T25_strip_geometry.csv` (the strip's near boundary per participant), `tables/T26_model_floor_sensitivity.csv` (the model's share with the box's floor lowered to the mat, computed on the main analysis itself — **not** the `turn0` variant of S3a, which is a per-frame check and also lowers the top surface)

S8. The confusion behind Section 3.2: what each rule assigned against each of the raters' seven labels, and why a rater could call the mat while the mapped point sat inside a printed region

Method. The raters' labels are crossed with each rule's assignment **before** the folding of Section 2.3, so that assignments to an area other than the one the rater's label belongs to are visible. Each rater's own covered and unclear items are dropped, as in S4 (171 items for rater 1, 176 for rater 2). The printed regions are working areas and are larger than the objects on them — the model's is printed at the size of the turntable base, 84.4 by 82.5 mm, and each tray's is 154 by 230 mm — so a point inside a printed region need not be on the object

Fixation set. The 200 sampled fixations of S4

Files. `tables/T23_confusion.csv` (rater × rule × rater label × assigned label)

S9. One row per participant, for the quantities the main text reports only as group medians or ranges

Method. Viewpoint height C_z and its distance C_y from the mat's origin; the horizontal distance D from that viewpoint to each object's printed centre and the planar displacement $D \cdot h/(C\_z - h)$ it implies; the number of analysed fixations and the share of the participant's fixations that reached the localized surface; the dwell share of the model and the copy under flat + 19 mm and under raised + 19 mm, and the height contribution that is their difference. **The Companion app's gaze offset correction**, applied only where the gaze marker was visibly displaced at the start of a session, is given per participant as recorded in each recording's `info.json` (`gaze_offset`, in scene-camera pixels) and as the angle it subtends at the image centre (S10e, `T32`)

Fixation set. The analysed set (4,798 fixations)

Files. `tables/T27_by_participant.csv` (with the offset in the last two columns)

S10. Six checks added at revision, on the same fixation table and region labels as the main analysis: (a) the two blocks separately; (b) other transition gap thresholds; (c) the accepted-pose subset by participant; (d) the excluded fixations by participant and block phase; (e) the gaze offset correction per participant; (f) the main contrasts by portion of the block and with the participants divided by surface coverage

Method. (a) Dwell shares under `P_m0`, `P_m19` and `F_m19` and the height and margin contributions computed per block instead of pooled, then summarised over the ten participants as in Table 2; the within-participant block 1 minus block 2 difference under `P_m19` is given as well. (b) The counting of Section 2.3 repeated with the gap between adjacent fixations limited to 0.5 s, 1 s (the reported value), 2 s and not limited at all. (c) For each participant the number of analysed fixations, the number with an accepted pose (time difference ≤ 100 ms, reprojection RMS ≤ 10 px), and on that subset the dwell share under the fixed viewpoint, under the per-frame pose, and their difference. (d) Each fixation inside a block placed in the first, middle or last third of that block by its onset; the fixations excluded from every region (not on the localized surface, 453 of 5,251) counted by participant and third. (e) `gaze_offset` read from each recording's `info.json`; the angle is atan(offset / focal length) with the scene camera's focal length (890 px), i.e. at the image centre. (f) The contrasts of Table 2 recomputed on the fixations in the first two

thirds of each block and on those in its last third (the thirds of (d)), and on the main analysis with the participants divided by their coverage over the blocks into the five lowest and the five highest

Fixation set. (a), (b) the analysed set (4,798 fixations); (c) the 2,498 with an accepted pose; (d) all 5,251 fixations inside the imitation blocks; (e) the ten recordings; (f) the 3,314 analysed fixations of the first two thirds (of 3,364 in them) and the 1,484 of the last third (of 1,887), and all 4,798 for the division by coverage

Files. `tables/T28_by_block.csv`, `T28b_by_block_summary.csv`, `T29_transition_gap.csv`, `T29b_transition_gap_by_participant.csv`, `T30_pose_subset_by_participant.csv`, `T31_excluded_by_phase.csv`, `T32_gaze_offset.csv`, `T33_by_block_portion.csv`, `T33b_by_block_portion_summary.csv`, `T34_by_coverage.csv`, `T34b_by_coverage_summary.csv`; produced by `code/revision.py`

Notes

- **S6 is a geometric calculation only.** It is not the result of recording with a new layout, and the existing fixations were not re-applied to the changed layout. The margin convention used throughout is: enlarge the printed footprint by 19 mm, then give it the object's height, then project — identical to the `F_m19` region of the main analysis. Projecting first and enlarging on the plane afterwards is a different rule and was not used.
- **S3a is computed on the dynamic method.** The height variants were generated by `assign.py` as `B_m19_*`. They bound how much the assumed heights matter for the per-frame box; they are not a sensitivity analysis of the fixed-viewpoint region used in the main text.
- **What S1 does not establish.** 19.0 mm is a single representative value from one wearer in one session. It is not a per-participant error for the ten participants of the main analysis, and it does not guarantee that any stated proportion of gaze falls within 19 mm.
- **Two of the eleven targets carry the `gaze_cal` role.** The mat's design file marks four fixation dots `gaze_cal` and twelve `gaze_eval`, and its own note says the `gaze_cal` dots are to be fixated in a separate calibration recording and excluded from the primary metric. The aggregation used here does not filter by that role: all eleven targets that met the selection criteria were pointed at in the same way within the same session, and the role recorded in the design file was not used to divide the measurement. Two `gaze_cal` targets (F20 and F23, errors 14.3 and 17.5 mm) are therefore among the 11. On the nine `gaze_eval` targets alone the median is **22.0 mm** rather than 19.0 mm; that figure is given so that a reader can see what the role-based split would produce. The manuscript uses 19 mm throughout. 22 mm lies inside the 14 to 24 mm range swept in S1b, over which the height contribution keeps its direction and its rough size. **This stability does not extend to the layout results.** The overlap of the projected regions and the move that clears it both grow with the margin — from 212 mm² and a 10 mm move at a 14 mm margin to 1,878 mm² and a 30 mm move at 24 mm (S6) — so a different margin would change the figures of Section 3.3 even where it leaves the height contribution intact.
- **What S1b does not establish.** Part (i) shows how far the height contribution moves over margins of 14 to 24 mm on these recordings: it is not evidence that 19 mm is optimal, that either region is the more accurate one, or that the pattern continues outside that range. Part (ii) and Figure 4 show what a wider margin costs in ambiguity on this layout with these viewpoints; the numbers are specific to both. Neither part selects a margin, and the layout move of Section 3.3 is not equally stable — the overlap and the clearing move both grow with the margin (S6).
- **S4 uses the raters' labels, not a ground truth.** The gaze marker on the sheets comes from the same eye-tracking estimate as the analysis, so a rater can state what the marker was over, not where the participant was looking; that is a weaker quantity than an independent record of the gaze target. Agreement between the two raters is the reproducibility of that judgement.
- **The agreement is with an area of interest, not with an object.** The printed footprint of the model is the turntable it stands on, and the copy's is the whole baseplate, so `BASE`→model and `PLATE`→copy follow from how the regions are drawn. Under that fold, 10.8% of the 195 items `F_m19` assigned to an object's area carried the label turntable or board and 6.7% bare baseplate (rater 2: 10.8% and 7.7%): inside the right area, not on the object. The three alternative folds are in `T18_interrater.csv` under their own `setting` values; the main text does not use the one that gives the highest agreement.
- **Two different exclusion rules, for two different quantities.** A comparison between a region and a rater can only use the items that rater judged, so it drops that rater's own covered and unclear items: 29 of the 200 for rater 1 and 24 for rater 2. A comparison between the two raters needs items both of them judged, so it drops the covered and unclear items of either, which is 33 of the 200. This is why Table 3 has denominators of 171 and 176 while the inter-rater agreement has 167. The reference figures apply neither rule to the covered items and drop only the unclear ones, giving 198 for each rater against the regions and 196 between the raters.
- **The covered items are set aside because the category becomes interpretive.** Where the marker sits on a hand, or on a brick the hand is holding, the rater has to separate the object the marker overlaps from the object behind it, and which area the item belongs to is then a matter of interpretation rather than of what is visible. The figures keeping those items are reported alongside, never on their own. This limits the scope of the check, and by a different amount for each comparison: of the 200 items, 29 fall outside rater 1's comparison with the regions, 24 outside rater 2's, and 33 outside the comparison between the two raters. Nothing in S4 speaks to how either region behaves while an object is being handled.

- **brick, handheld, moving and borderline flags are not part of S4.** The 200 items were stratified to check which area a fixation fell in, not to estimate how often an object was being moved, so rates of that kind cannot be taken from this sample. One item in rater 1's table carries a borderline flag, which is not part of the label set; the aggregation ignores it, and treating it as covered changes no reported figure (167 → 166 items, agreement 0.982, κ = 0.975 either way).
- **The 91.4% on-surface share is not uniform across participants.** By participant it runs 73.3, 81.9, 83.2, 87.4, 95.3, 95.6, 96.6, 98.6, 99.2 and 100.0%. The lowest belongs to the participant whose model share moves most under height (Section 4.2), which is why that case is discussed there rather than treated as a group figure. S10f divides the participants at the middle of this list, the lowest five against the highest five.
- **What S10 shows.** (a) Analysed block by block, the two imitation blocks give the picture of Table 2 twice over. The copy's height contribution is +1.6 points [0.1, 9.3] in block 1, positive in all ten participants, and +1.1 [0.0, 11.3] in block 2, positive in nine and exactly zero in one; the model's is +1.5 [−10.1, +30.5] and −0.2 [−2.7, +33.5], five participants against five in both blocks; the unassigned share changes by −5.4 [−40.0, +1.7] and −4.4 [−45.0, +2.5], falling in eight of ten in both. The margin contributions to the model, the copy and the unassigned share keep their sign in all ten participants in both blocks (copy +5.7 and +4.6, model +9.4 and +8.2, unassigned −15.9 and −15.5); the trays' is positive in all ten in block 1 and in eight in block 2, the other two unchanged. Block 2 was shorter in eight of ten participants (median 136 s against 122 s), and under flat + 19 mm the shares themselves moved a little between blocks (model +2.6 points [−5.4, +7.7] in block 1 relative to block 2, tray −1.7 [−12.0, +1.1], with nine of ten looking at the trays more in block 2). Something therefore changed between the blocks, whether through practice, fatigue or the layer order of the model, and this comparison cannot separate these; the group summaries of what the choice of region did to the numbers did not change, while within a participant a contribution can differ between the blocks by up to 12 points (the margin's contribution to the model is 26.6 and 14.2 for one participant, the model's height contribution −10.1 and +0.1 for another; T28). (b) The transition counts of Section 3.3 are identical at 0.5 s, 1 s, 2 s and with no threshold: of the 4,737 evaluable adjacent pairs, 41 have a gap above 0.5 s, 13 above 1 s and 3 above 2 s, and none of them is a model-to-copy or tray-to-copy pair under any of the three regions compared, so every rate and within-participant difference in Section 3.3 is the same at every threshold. (c) The accepted-pose subset holds 52 to 450 fixations per participant, 10.4% to 98.3% of that participant's analysed fixations, with two participants below 40% (35.3% and 10.4%); on it the per-frame pose moves a dwell share from the fixed viewpoint by at most 2.5 points (one participant, model +2.5 and tray −2.5), by 1.1 points in one other (copy and unassigned), and by 0.2 points or less in the remaining eight. (d) Of the 453 fixations inside the blocks that were not on the localized surface, 403 (89.0%) lie in the last third of a block. Five participants account for 407 of the 453 (25, 92, 54, 123 and 113 fixations), 397 of them in the last third, where they make up 17% to 68% of that third's fixations; of the remaining 46, most fall in the first third of three participants' blocks (16, 5 and 15 fixations), one participant has none, and the middle thirds of all ten hold 6 in total. The excluded fixations are therefore not spread over the task but sit at the ends of blocks. (e) The gaze offset correction was applied in six of the ten recordings, by 5.7, 15.7, 21.2, 42.7, 63.7 and 96.8 px in the scene camera (0.37°, 1.01°, 1.36°, 2.75°, 4.09° and 6.21° at the image centre), and not at all in the other four; all ten were recorded with the same Companion app (2.9.47-prod), gaze pipeline (2.9.0) and firmware (29.8). The correction is applied by the app to the gaze estimate as it is recorded, so every analysis in this paper already contains it. It records how far the gaze was shifted, not how much error remained afterwards, so these values are not a measure of any participant's accuracy and are not used as one. (f) On the first two thirds of each block, which hold 3,364 of the 5,251 fixations in the blocks and 50 of the 453 that did not reach the surface (coverage 94.3% to 100% per participant, median 99.1%; T33, T33b), the margin's contribution remains the larger at the group median: copy +5.9 points [1.0, 22.9] against +0.4 [0.0, 7.7] for height, unassigned −13.2 [−29.2, −5.5] against −2.3 [−42.6, +2.3]. The margin's contribution keeps its sign in all ten participants for the model, the copy and the unassigned share, and the trays' is positive in eight, the other two unchanged; the model's height contribution splits five against five (−0.3 [−7.1, +34.7]); and the copy's is positive in eight participants and zero in two, none negative. On the last third (coverage 31.7% to 100%, median 90.5%) the copy's height contribution is +4.7 [+0.3, +24.5], positive in all ten, so much of the copy's gain under height comes at the end of the blocks, when the copy is close to the height of the proxy (Section 2.1); the model rises in seven participants and falls in three there. Divided by their coverage over the blocks (T34, T34b), the five participants with the lowest coverage (73.3% to 95.3%), who are the five of (d) with 407 of the 453 excluded fixations, and the five with the highest (95.6% to 100%) both show the margin's contribution as the larger at the group median (copy +4.8 against +1.1 in the lower group, +9.3 against +3.7 in the upper) and the copy's height contribution positive in all five. The model's height contribution rises in one participant and falls in four in the lower group (−1.8 [−5.7, +32.0]) and rises in four and falls in one in the upper (+5.3 [−0.5, +16.7]), the largest rise belonging to the participant with the lowest coverage. With five participants in a group these are descriptions, not tests, and every share is a share of the fixation time that reached the surface.
- **S2 and S3a are computed on the subset they describe.** In the submitted version T11 and T10 had been produced from the primary set, on which the per-frame box takes the nearest recovered pose whatever its time difference; they are now produced from the 2,498 fixations with an accepted pose, as S2 and S3a state. In S2 the largest within-participant difference is 2.5 points (it was 3.5 under the earlier computation); in S3a the largest change in any participant is 18.7 points (it was 14.0) and the largest group median 0.8 points (it was 1.8), both on the model in the same variant. The correction affects no other table.
- Everything above is produced by the code in `code/` from the files in `data/`; see `README.md` for the single command sequence that regenerates it.
